# Effect of productivity and seasonal variation on phytoplankton intermittency in a microscale ecological study using closure approach

**DOI:** 10.1038/s41598-022-09420-5

**Published:** 2022-04-08

**Authors:** Arpita Mondal, Sandip Banerjee

**Affiliations:** Department of Mathematics, Indian Institute of Technology Roorkee, Roorkee, Uttarakhand 247667 India

**Keywords:** Ecological modelling, Marine biology

## Abstract

A microscale ecological study using the closure approach to understand the impact of productivity controlled by geographical and seasonal variations on the intermittency of phytoplankton is done in this paper. Using this approach for a nutrient–phytoplankton model with Holling type III functional response, it has been shown how the dynamics of the system can be affected by the environmental fluctuations triggered by the impact of light, temperature, and salinity, which fluctuate with regional and seasonal variations. Reynold’s averaging method in space, which results in expressing the original components in terms of its mean (average value) and perturbation (fluctuation) has been used to determine the impact of growth fluctuation in phytoplankton distribution and in the intermittency of phytoplankton spreading (variance). Parameters are estimated from the nature of productivity and spread of phytoplankton density during field observation done at four different locations of Tokyo Bay. The model validation shows that our results are in good agreement with the field observation and succeeded in explaining the intermittent phytoplankton distribution at different locations of Tokyo Bay, Japan, and its neighboring coastal regions.

## Introduction

Phytoplankton is the major biomass producers in marine ecosystems which provides an essential ecological sustainability for all aquatic life. It accounts for nearly half of the total production of organic matter on Earth via photosynthesis, which is the fundamental biological process that converts inorganic carbon into living biomass. Through photosynthesis, productivity of phytoplankton fuels marine food web. The efficiency and dynamics of productivity influence the energy supply to higher trophic levels. The productivity of phytoplankton determined from the nature of photosynthesis is influenced by several biotic and abiotic factors, such as light^1^ (whose impact on phytoplankton productivity varies inversely with depth of water column), temperature^2^, salinity^2^ and many more. Environmental fluctuations triggered by the variation of these factors influenced by the impact of unpredictable radiation, climate change, variation in depth, which appear with regional and seasonal variations, causes oscillatory impact in the rate of photosynthesis, hence, phytoplankton productivity fluctuates. As phytoplankton productivity fluctuates, it causes oscillation in phytoplankton biomass. The composition and the biomass of the phytoplankton assemblage can be altered by the shifts in nutrient concentrations and ratios^3^. Hence, it is increasingly important to reevaluate the role of nutrient in controlling phytoplankton biomass as well as to understand the seasonal and spatial variation in phytoplankton.

It has been observed that Phytoplankton density is not distributed in a uniform manner, it varies with space and time^4^. This happens due to the impact of variation in temperature, salinity and water depth on the rate of photosynthesis, which results in fluctuation of phytoplankton density. Also, the system regains nutrient from dead plankton. Now, fluctuation in plankton density causes fluctuation in the death rate of these species, as a result of which the nutrient density also fluctuates.

The evaluation of spatial structure of phytoplankton distribution is generally centered around experimental observations at the scale of greater than 10 m to several kilometers, where inconsistency in phytoplankton distribution were empirically observed to be highly variable in both space and time^5^. The study of seasonal variation of nutrient and dynamics of plankton in aquatic systems was done by many researchers over the past few decades and mathematical models have been used as an essential tool for better mathematical understanding of these systems^6–9^. In both of these cases, the models are studied in mesoscale or in larger scale and very few in microscales. In contrast to large scale studies, there is limited information on the spatial optimization of phytoplankton at those ecologically relevant microscales.

The exploration of microscale structure of plankton in the oceans has been a technologically limited enterprise^10^. For example, collection of samples using Niskin bottle (which is used to take water samples at a desired depth without the danger of mixing with water from other depths) and Seapoint Chlorophyll Fluorometer (SCF, which is a high performance, low power instrument for in situ measurement of chlorophyll) have their limitations. Both of these instruments are not capable of collecting high-resolution microstructure fluorescence data. However, with improved technology, phytoplankton measurements of micro-scale distributions are done using a variety of high resolution instruments^11–20^, namely, Light Emitting Diode Sensor and Laser Sensor. Use of TurboMAP-L^11^, a free falling microstructure profiler that captures the high resolution data of undisturbed fields using laser sensor is the motivation of this study. One can also use FluoroMAP and FIDO-$\phi$ to capture high resolution data. These sophisticated high-resolution instruments are capable of various fluorescence based measurement systems, which captures highly resolved profiles of phytoplankton concentration and fluorescence and helps in better understanding of the dynamics of phytoplankton by giving better estimate of model parameters^21^. They advance our comprehension of the impact of physical processes on plankton dynamics, like irregular distribution or intermittency, irregularity in nutrient load pattern, variation in the production of global carbon cycling related to marine ecosystem, spring blooms (surges of phytoplankton population occur seasonally), red tides (localized outbreaks of phytoplankton population), from mesoscale to microscale. The availability of the high resolution data of undisturbed fields creates a motivation of modelling the nutrient–phytoplankton dynamics at the microscale level using moment closure approach to include the contribution of high-resolution microscale data in the formulated model, which helps to better capture and explain the observed scenario.

Moment closure method is applied in terrestrial ecology to consider the effects of dynamical nonlinearity acting on spatial variability. To capture the contribution of growth fluctuation in phytoplankton distribution and for understanding the intermittency in phytoplankton spreading (variance), we have used Reynold’s averaging method in space. This method helps in writing the original components in terms of its average values (mean) and fluctuation or perturbation. Generally this method is applicable to temperature and wind velocity in turbulent flow, but can also be considered in any system where turbulent dynamics are present. Mandal et al.^22^ have used the moment closure approach for a simple nutrient–phytoplankton model and showed the effect of fluctuating parts of model variables on the system dynamics. Priyadarshi et al.^23^ also used the same technique to study the micro-scale spatial variability in a nutrient–phytoplankton–zooplankton model. Both of them used a spatially explicit second order moment approximation for the variance and covariance of each state variable. It is to be noted that the mean fields are quantified at the meter-scale, whereas the fluctuating fields are quantified at the millimeter-scale. The second order moments have impacts on the dynamics of mean field due to explicit equations for the rate of change of means, variances and covariances.

On the basis of observations of phytoplankton collected at the mouth of Tokyo Bay, Japan (high resolution data), showing the importance of considering the fluctuating part of each component of the system as a new variable, we aim to study the impact of this variability, namely, the intermittency of phytoplankton, on the output of the model and then compare its dynamics with real observations. Though a similar study can be seen in^22^, but we indulge in a much deeper investigation of plankton intermittency with the help of a new NP-model. To estimate the parameter values of the model, more aspects that influence environmental factors like sunlight, uv radiation, water temperature, salinity, nature of dominating phytoplankton species, variation in density of chlorophyll, which play major role in phytoplankton productivity are considered, including the seasonal and regional influences of these factors. Better estimates of the parameter values are obtained considering all these factors, which properly explains the overall nature of phytoplankton productivity, one of the most important reason behind this irregularity in regional and seasonal phytoplankton distribution.

## Nutrient–phytoplankton closure model

At the initial stage, we concentrate only on nutrient (*N*) and phytoplankton (*P*) as model variables in order to develop the methodology of a new ocean ecosystem model. As discussed before, factors like, light^1^, temperature^2^ and salinity of water^2^ make it difficult to determine a clear mechanism of phytoplankton distribution through experimental observations in the case of real aquatic environment. Not all these natural phenomena are governed by deterministic laws, instead they oscillate randomly about some average behaviours being influenced by unpredictable radiation, climate change and variation in water depth. Such environmental fluctuations disturb the rate of photosynthesis, as a result of which maximum growth rate of phytoplankton oscillates in a range and accordingly death rate varies. Hence, growth fluctuation and variation of mortality of phytoplankton cause fluctuation in the density of nutrient and phytoplankton. Therefore, while formulating the NP model, we consider two parameters regarding the growth and death rate of phytoplankton, which will be determined by the nature of its productivity with regional and seasonal variations. Due to the presence of intermittency in phytoplankton distribution^22^ triggered by the presence of fluctuation in the biomass, we also consider fluctuating terms or perturbations of the density of nutrient and phytoplankton about their mean values, so that the irregularity that is experimentally observed in phytoplankton distribution can also be captured while formulating the model.

Thus, the NP model with Holling type III functional response and linear mortality of phytoplankton is given by,

1 $$\begin{aligned}\dfrac{dN}{dt}&=-C\frac{N^{2}P}{K^{2}+N^{2}}+DP,\nonumber \\\dfrac{dP}{dt}&=C\frac{N^{2}P}{K^{2}+N^{2}}-DP, \end{aligned}$$

where *C* is the maximum growth rate of phytoplankton, which depends on the intensity of light, *D* is phytoplankton death rate (mortality rate) and *K* is half-saturation constant. The phytoplankton functional response is usually defined as the specific food intake rate (i.e. per phytoplankton biomass, per unit of time) as a function of the ambient food density. We assume that the functional response of NP-model is of sigmoid type and density-dependent, which works properly with many models of phytoplankton blooms^24–27^. Hence, we have chosen Holling type III functional response to formulate our model. Since the observations were done at different sampling stations during different time periods, dominating phytoplankton communities vary differently for all regions. Depending on climate change and due to regional variations, nutrient density also varies. Since this variation of nutrient density is an important factor through out the field observation, we need to consider a functional response that will take this factor into consideration and will provide some realistic mechanism of model variables.

The system is conserved, that is, $N+P=A\, ({\text {constant}})$. During the studies of microscale phytoplankton distribution using high-resolution profiling fluorometers, it has been observed that the local values of fluorescence are highly fluctuating in space^11,28^. Since the biomass of phytoplankton in the system is interrelated to the amount of nutrient, it is expected to be fluctuating in space for a closed system. However, non-closure model as described by Eq. (1) does not consider this spatial variability.

## Methods

To derive the NP-closure model from the conventional NP model (non-closure), the model variables are considered to be a function of both time (*t*) and space (*r*), namely,

2 $$\begin{aligned} P(r,t)=P_{0}(r,t)+ P^{\prime}(r,t)\,\,\,{\text {and}}\,\,\,N(r,t)=N_{0}(r,t)+ N^{\prime}(r,t), \end{aligned}$$

where $N_{0}$, $P_{0}$ are spatial mean values of the nutrient and phytoplankton respectively and $N^{\prime}$, $P^{\prime}$ are their respective fluctuating components corresponding to each mean value. Please note that the fluctuating part is not a stochastic component here but it is actually a perturbation around the average value of the state variable. Hence, the fluctuating part does not possess any stochastic properties. However, due to the impact of environmental phenomena this fluctuation/perturbation has been introduced to the system. The horizontal and vertical sampling for microscale phytoplankton distribution have the same statistics at the centimeter scale^29^, also at the millimeter scale (except for extreme values). Hence, the statistics of the fluctuating components is independent of the direction of sampling (isotropic). Therefore, the spatial average of each fluctuating component is zero at any particular time, that is $\langle P^{\prime}(r)\rangle =0$, $\langle N^{\prime}(r)\rangle =0$, while its temporal average cannot be zero, which implies $\langle P(t)\rangle =P_{0}(t)$ and $\langle N(t)\rangle =N_{0}(t)$. Substituting (2) in (1) (applying the Reynold’s averaging method in space) and retaining only up to second order terms in Taylor series expansion, we obtain the following set of equations for the closure model as,

3 $$\begin{aligned} \frac{dP_0}{dt}&=\frac{C N_0^{2} P_0}{(K^2+N_0^{2})}+\frac{2CK^2 N_0 \langle N^{\prime}P^{\prime}\rangle }{(K^2+N_0^{2})^2} -D P_0 + \frac{CK^2 P_0 \langle N^{\prime 2}\rangle (K^2-3N_0^{2})}{(K^2+N_0^{2})^3}\end{aligned}$$

4 $$\begin{aligned} \frac{dN_0}{dt}&= -\frac{C N_0^{2} P_0}{(K^2+N_0^{2})}-\frac{2CK^2 N_0 \langle N^{\prime}P^{\prime}\rangle }{(K^2+N_0^{2})^2} +D P_0 -\frac{CK^2 P_0 \langle N^{\prime 2}\rangle (K^2-3N_0^{2})}{(K^2+N_0^{2})^3} \end{aligned}$$

5 $$\begin{aligned} \frac{d\langle P^{\prime 2}\rangle }{dt}&= \frac{2CN_0^{2}\langle P^{\prime 2}\rangle }{(K^2+N_0^{2})}+\frac{4CK^2N_0P_0\langle N^{\prime}P^{\prime}\rangle }{(K^2+N_0^{2})^2}-2D\langle P^{\prime 2} \rangle \end{aligned}$$

6 $$\begin{aligned} \frac{d\langle N^{\prime 2}\rangle }{dt}&= -\frac{2CN_0^{2}\langle N^{\prime}P^{\prime}\rangle }{(K^2+N_0^{2})}-\frac{4CK^2N_0P_0\langle N^{\prime 2}\rangle }{(K^2+N_0^{2})^2}+2D\langle N^{\prime}P^{\prime}\rangle \end{aligned}$$

7 $$\begin{aligned} \frac{d\langle N^{\prime}P^{\prime}\rangle }{dt}&= \frac{CN_0^{2}(\langle N^{\prime}P^{\prime}\rangle -\langle P^{\prime 2}\rangle )}{(K^2+N_0^{2})}-D(\langle N^{\prime}P^{\prime}\rangle -\langle P^{\prime 2}\rangle ) +\frac{2CK^2N_0P_0(\langle N^{\prime 2}\rangle -\langle N^{\prime}P^{\prime}\rangle )}{(K^2+N_0^{2})^2} \end{aligned}$$

We have assumed that the random variables ${\mathbf {N}}$ and ${\mathbf {P}}$ follow a joint lognormal probability distribution whose observed values are *N*, *P* (densities of phytoplankton and nutrient respectively). The lognormal distribution can be fitted well to empirical data^30^ and has been widely used in continuous model^31–33^. We have ignored the third and higher order fluctuating terms to obtain simple closure.

Equations (3) and (4) represent the time evolution of mean terms, Eqs. (5) and (6) represent time evolution of variance terms and Eq. (7) gives the time evolution of covariance term. Adding Eqs. (3) and (4), we obtain

$$\begin{aligned} \frac{dN_0}{dt} +\frac{dP_0}{dt}=0\Rightarrow N_0+P_0=A\,({\text {constant}}). \end{aligned}$$

Again, adding (5), (6) and (7) we obtain,

$$\begin{aligned}&\frac{1}{2}\frac{d\langle N^{\prime 2}\rangle }{dt}+\frac{1}{2}\frac{d\langle P^{\prime 2}\rangle }{dt}+\frac{d\langle N^{\prime}P^{\prime}\rangle }{dt}=0\\&\quad \Rightarrow \langle N^{\prime 2}\rangle +\langle P^{\prime 2}\rangle +2\langle N^{\prime}P^{\prime}\rangle =B\,({\text {constant}})\Rightarrow B= \langle (N^{\prime}+P^{\prime})^{2}\rangle , \end{aligned}$$

where *B* is the variance of the sum of $N^{\prime}$ and $P^{\prime}$. Therefore, both $N_0+P_0$ and $\langle N^{\prime 2}\rangle +\langle P^{\prime 2}\rangle +2\langle N^{\prime}P^{\prime}\rangle$ are temporary conserved quantities.

With appropriate scaling, the five equations given by (3)–(7) can be reduced to three equations with three dimensionless parameters (Table 1):

8 $$\begin{aligned}&\frac{dp_0}{d \tau }=\frac{(1-p_0)^{2}p_0}{(k^2+(1-p_0)^{2})}+\frac{k^2(1-p_0)(\beta -x-y)}{(k^2+(1-p_0)^{2})^2} +\frac{k^2p_0(k^2-3(1-p_0)^{2})y}{(k^2+(1-p_0)^{2})^3}-\varepsilon p_0, \end{aligned}$$

9 $$\begin{aligned}&\frac{dx}{d \tau }=\frac{2(1-p_0)^{2}x}{(k^2+(1-p_0)^{2})}+\frac{2k^2(1-p_0)p_0(\beta -x-y)}{(k^2+(1-p_0)^{2})^2}-2\varepsilon x, \end{aligned}$$

10 $$\begin{aligned}&\frac{dy}{d \tau }=-\frac{(1-p_0)^{2}(\beta -x-y)}{(k^2+(1-p_0)^{2})}-\frac{4k^2(1-p_0)p_0y}{(k^2+(1-p_0)^{2})^2}+\varepsilon (\beta -x-y),\nonumber \\&\quad {\text {with}}\,\,\, n_0+p_0=1,x+y+2z=\beta \end{aligned}$$

where $\beta$, the normalized sum of variance and covariance, is given by,

$$\begin{aligned} \beta =\frac{\langle N^{\prime 2}\rangle +\langle P^{\prime 2}\rangle +2\langle N^{\prime}P^{\prime}\rangle }{A^2}=\frac{\langle (N^{\prime}+P^{\prime})^2\rangle}{A^2}=\frac{B}{A^2}, \end{aligned}$$

which represents a standardized measure of total variability in the closure model, where *B* is the variance of sum of fluctuating components $N^{\prime}$ and $P^{\prime}$, *A* is the sum of the spatial mean values of the nutrient and phytoplankton. Hence, $\beta$ is a overall variation of the system.

**Table 1:** Salinity versus depth in Tokyo Bay during Spring (collected from^34^).

Depth (m)	Southern part (Region 1) (PSU)	Central part (Region 2) (PSU)	Northern part (PSU)
3	32.4	30.2–32	< 30.2
10	< 32.9	32.0–32.8	32.2
15	< 33.2	33.2–33.6	33.4

All observations at different sampling stations of Tokyo Bay during 2006–2011 are connected to coefficient of variation of phytoplankton biomass ($CV_P$), which is actually a measurable quantity. The coefficient of variation is defined as the ratio of standard deviation to the mean of the variable, which gives a normalized measure of variability of a distribution. For this model it is expected to determine the coefficient of variation as the closure model considers the fluctuation of variables. The coefficient of variation for phytoplankton can be greater than or less than 1 depending on the parameter values^22^. Now, environmental heterogeneity can be defined as

$$\begin{aligned} \beta =\frac{\sum {\mathbf {Variance}}+2\times {\mathbf {Covariance}}}{{\mathbf {Mean}}^{\mathbf{2}}}. \end{aligned}$$

The whole analysis is based on the nature of values of $\beta$ and on the nature of the ratio of S.D ($\sqrt{x}$) and mean ($p_0$) of phytoplankton since we know,

$$\begin{aligned} {CV_P}=\frac{\sqrt{\langle P^{\prime 2}\rangle }}{P_0}=\frac{\sqrt{A^{2} x}}{A p_0}=\frac{\sqrt{x}}{p_0}. \end{aligned}$$

**Figure 1:**
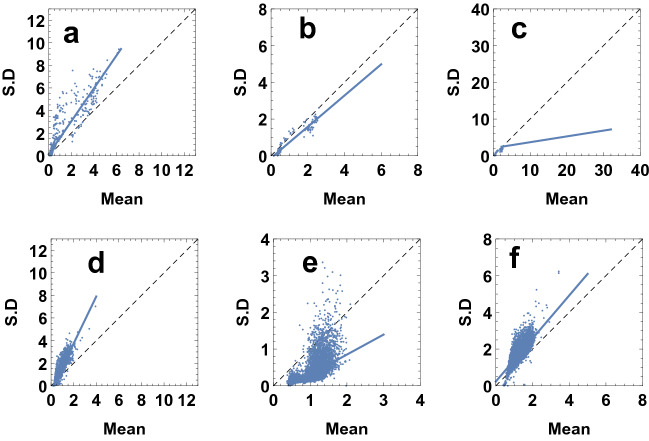
Observations of phytoplankton data in Tokyo Bay. Figures (**a**, **b**, **c**) show mean and standard deviation of fluorescence microstructure obtained from different locations in Tokyo Bay in May 2011. Figures (**d**, **e**, **f**) show data obtained from same location in Tokyo Bay but at different months (collected from^46^).

Based on the obtained dynamics of variables and nature of calculated $CV_P$, we will take into consideration the most suitable fact which resonates with the nature of measured $CV_P$ (Fig. 1a–f).

Please note that this process can be repeated for any number of collected samples as we have assumed that the population of nutrient and phytoplankton follows joint lognormal probability distribution^30–33^. Therefore, samples taken from any part of the population will follow the same distribution, which is true for the samples collected at the mouth of Tokyo Bay (Region 1), inside Tokyo Bay (Region 2) and the mouth of Arakawa river (Region 3). Hence, different data sets with same procedure will not produce contradictory results.

## Estimation of system parameters

The closure model under consideration contains three unknown parameters, namely, *C* (maximum growth rate of phytoplankton), *K* (half-saturation constant) and *D* (linear mortality rate of phytoplankton). Experiments were conducted and data were collected from four different locations of Tokyo Bay during different seasons^22^. In May 2011, the field observations were done at three different locations of Tokyo Bay:

(i) Region 1: the mouth of Tokyo Bay,
(ii) Region 2: inside Tokyo Bay,
(iii) Region 3: the mouth of Arakawa river.

From these locations microstructure data were collected from the depths of approximately 200 m, 50 m and 10 m respectively. In these cases, samples were collected from three different stations in the month of May, 2011 (no seasonal variations, Fig. 1a,b,c). To observe the impact of seasonal variation on phytoplankton distribution, fluorescence data were collected from the same depth ($\approx 50$ m) of a particular location (Region 4: inside Tokyo Bay but different from Region 2) during three different months, namely, December 2006, September 2007, February 2008 (Fig. 1d,e,f).

Figure 1 indicates different kind of phytoplankton distributions at different euphotic zones of Tokyo Bay during different times and these differences in distribution are caused by change in rate of photosynthesis of phytoplankton. From observational data obtained from three different depths of Region 1, Region 2 and Region 3 during the same period, we observe that there is irregularity in phytoplankton distribution at all these regions and this kind of irregularity is caused by differences in productivity, growth and mortality of phytoplankton, which vary with zonal variations. Since photosynthesis is mostly dependent on water-temperature and salinity, we first discuss how water-temperature and salinity have varied for three different regions in the month of May, 2011 and then we discuss about the possible changes in phytoplankton growth and mortality during that time for each individual region at Tokyo Bay.

**Figure 2:**
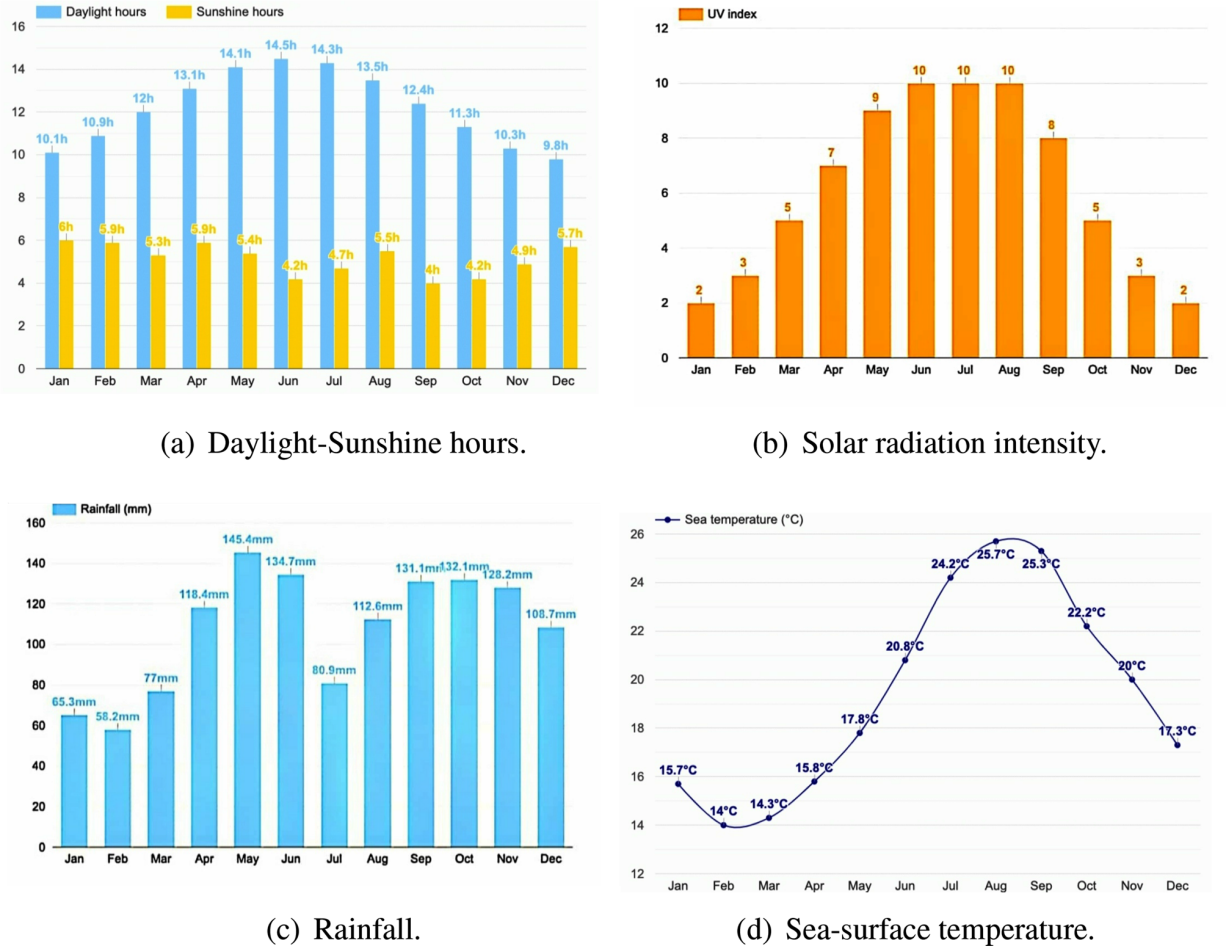
(**a**) Monthly daylight sunshine hours per day. (**b**) Average uv-index corresponding to every month inside and around Tokyo Bay, Japan. (**c**) Monthly average rainfall. (**d**) Sea-surface temperature inside and around Tokyo Bay (collected from^38^).

During the month of May, average daylight and sunshine hours inside and around Tokyo Bay are 14.1 h and 5.4 h respectively (Fig. 2a), which are quite high. Figure 2b indicates that the intensity of solar radiation (UV), which usually remains 9 (UV index) in the month of May. These factors influence sea-surface temperature to increase a lot, which is also affected by the occurrence of rainfall during this month (Fig. 2c). As a result, during this time average sea-surface temperature remains $17.8\,^{\circ }$C (Fig. 2d) at Region 1 (mouth of Tokyo Bay) and Region 2 (inside Tokyo Bay), which decreases with increasing depth.

### Region 1: Mouth of Tokyo Bay

**Figure 3:**
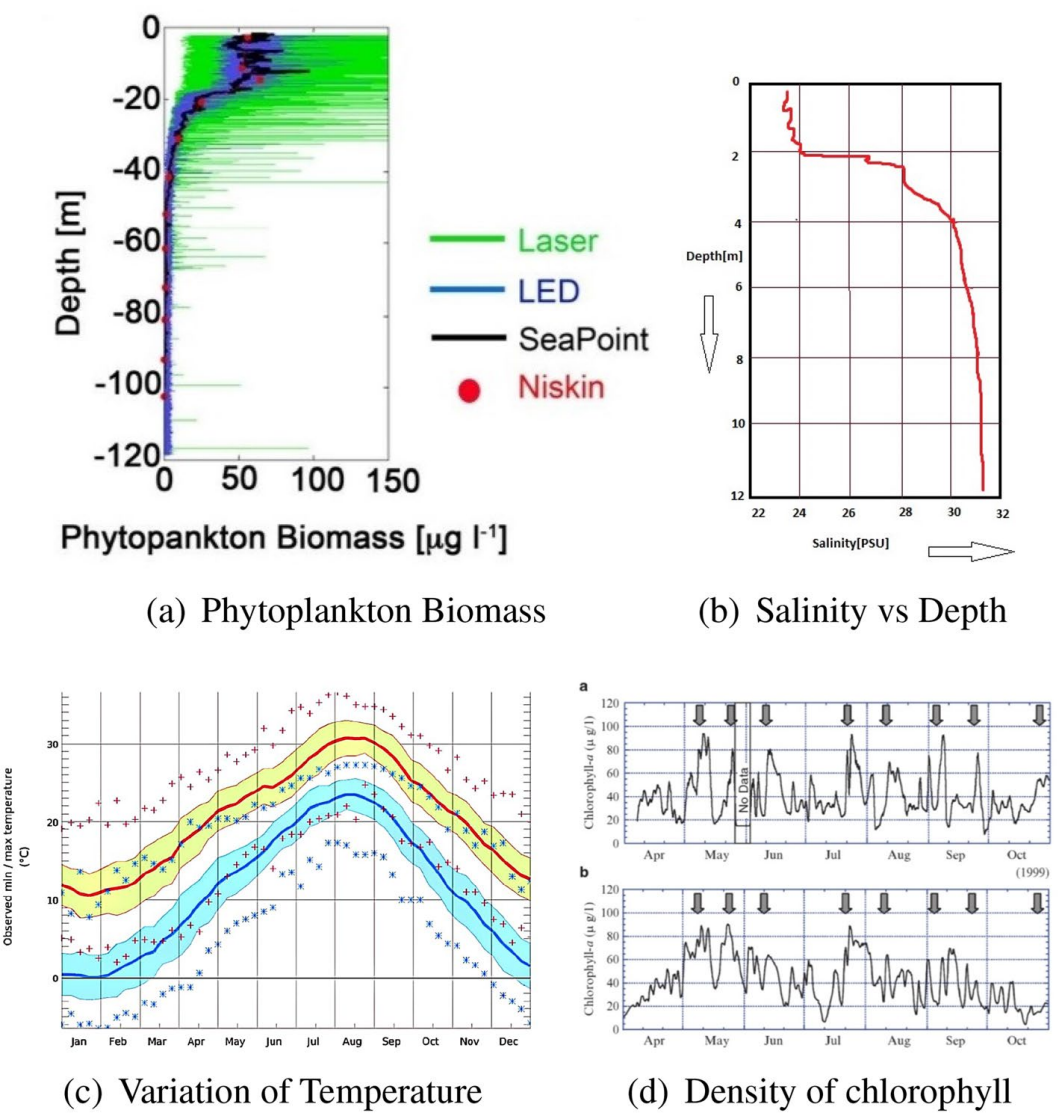
(**a**) Plot of data at different depths at the mouth of Tokyo Bay(region 1) on June 18, 2011^22^. (**b**) Salinity observed on 19th May 2011 at the mouth of Arakawa river (collected from^47^). (**c**) Variation of temperature with seasons according to observed data near the mouth of Arakawa river (collectecd from^48^). (**d**) Chlorophyll concentration versus month in the innermost part of Tokyo Bay which includes Region 2 and Region 4 (collected from^40^, Figs. 3–9).

Data regarding mean and standard deviation (SD) of phytoplankton were obtained from the observational data collected from a depth of nearly 200 m at Region 1. Figure 3a indicates that as depth increases, density (after 60 m) of chlorophyll gradually becomes low. During the month of May, climate condition is favourable for optimum level of photosynthesis of phytoplankton on the upper layer of sea mostly up to the depth of 60 m as a result of which, as depth increases chlorophyll density decreases rapidly, which have a negative impact on photosynthesis. According to Fig. 2d, sea-surface temperature of Tokyo Bay during the month May remains $17.8\,^{\circ }$C, which is good for photosynthesis of phytoplankton but at the depth of 200 m, intensity of light and solar radiation, both are very low and hence temperature remains quite low, which reduces the rate of photosynthesis and slows down the growth of phytoplankton.

Region 1 belongs to southern part of the bay, which is open to Pacific ocean. Since a portion of ocean water mixes with the water of these regions, salinity of this zone remain higher than that of northern part of Tokyo Bay (Table 1). Thus, we conclude that salinity remains very high at the depth of 200 m. However, lack of temperature, sunlight, chlorophyll hinder the productivity of phytoplankton at that depth of Region 1 in May, 2011, even though salinity is high. This ultimately results in low value of *C* (which varies inversely with depth). Since *C* is low( $\approx$ 0.2/day^22^), mean of phytoplankton is also low there. Since $P_0$ is low at that depth (200 m), water is enriched with high density of nutrient $N_0$ (since NP system is closed), mortality rate of phytoplankton (*D)* should be higher at that depth than the sea-surface level. Overall, range of phytoplankton death rate (*D*) is (0.07–0.2)/day^22^. Thus,

$$\begin{aligned} 0.07\le D\le 0.2\Rightarrow \frac{0.07}{C}\le \frac{D}{C}\le \frac{0.2}{C}. \end{aligned}$$

At the depth of 200 m of Region 1, $C\approx0.2$^22^ which implies

$$\begin{aligned} \frac{0.07}{0.2}\le \frac{D}{C}\le \frac{0.2}{0.2}\Rightarrow 0.35 \le \varepsilon \le 1. \end{aligned}$$

Hence, $\varepsilon \left( =\frac{D}{C} \right)$ will have the range (0.35, 1). Since, in the case of field observation, the experimental depth of this region is the highest of all other depths of all other considered zones, maximum growth *C* should be the very low at that depth compared to the value of *C* on upper sea surface of this same location (Region 1). Though, due to low productivity, overall range of $\varepsilon$ is (0.35, 1.0) but for having maximum depth, values of $\varepsilon$ (since $\varepsilon \varpropto \frac{1}{C}$), which will determine the dynamics of variables for this region, should be closer to 1.

### Region 2: Inside Tokyo Bay

In real observations, microstructure fluorescence data was collected from Region 2 in May, 2011 from a depth of nearly 50 m. Figure 3d indicates high chlorophyll density at Region 2 in the month of May. Table 1 indicates that as depth increases salinity increases, and at that depth of 50 m salinity remains $\ge 33.6\,{\text {PSU}}$. Since this kind of salinity and temperature is suitable for photosynthesis, maximum growth of phytoplankton (*C*) remains high $\left( \approx 2\,{\mathrm{day}}^{-1}\right)$. Since *C* is high (depth is comparatively low), mean of phytoplankton is also high there $\left( N_0+P_0=A= {\text {constant}}\right)$. Also, $P_0$ is high at that depth (50 m) and water is enriched with low density of nutrient $N_0$ (since NP system is closed), mortality rate *D* of phytoplankton should be low at that depth. Overall, region of *D* is (0.07–0.2) ${\mathrm{day}}^{-1}$ and $C\approx 2$^22^, hence, overall range of $\varepsilon \left( =\frac{D}{C}\right)$ will be (0.035, 0.1). Region 2 is near to station F7 and station O2, where in and around these stations, dominating phytoplankton class is mainly diatom and among all diatom classes, *Skeletonema Costatum* is the dominant class^35^. Same fact is true for Region 4.

### Region 3: Mouth of Arakawa river

Data was collected from the depth of nearly 10 m at the mouth of Arakawa river^22^, where the water temperature near the mouth remains (9–$27)\,^{\circ }$C (Fig. 3c). Since sunlight hits directly the uppermost layer of sea and intensity of light is high there, we assume temperature remains (25–$27)\,^{\circ }$C at that depth. This high temperature increases the rate of photosynthesis ($20\,^{\circ }$C is best for photosynthesis of phytoplankton on euphotic zone of sea whereas temperature above $40\,^{\circ }$C slows down its rate), which results in maximum *C*. At the mouth of Arakawa river (Region 3) during May 2011, salinity of surface water in (0–2 m) was (22–24) ${\text {PSU}}$ but as depth increases, salinity of water also increases and lies between (30–32) ${\text {PSU}}$ at the depth of 10 m (Fig. 3b).

**Figure 4:**
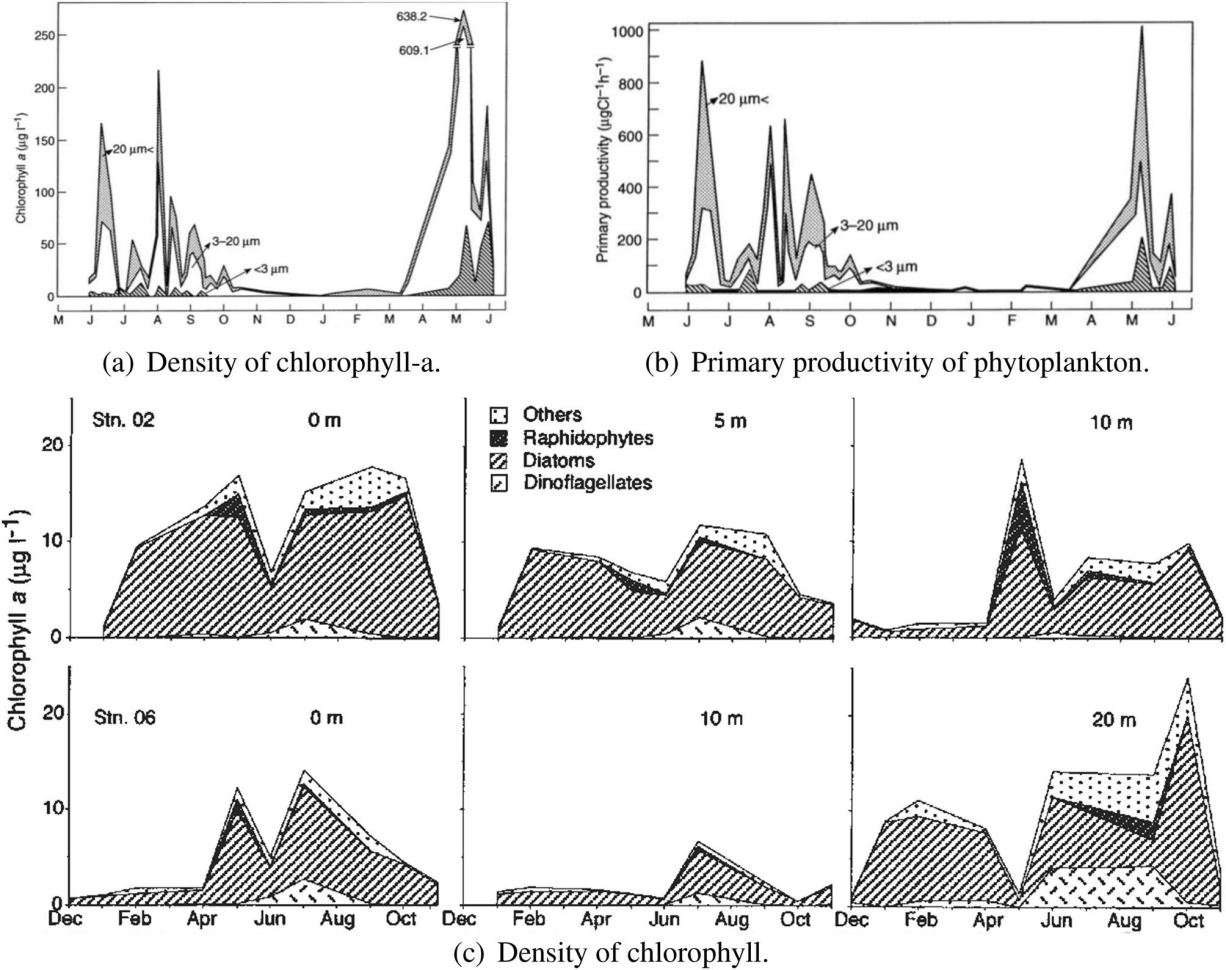
(**a**) Monthly variation in concentration of chlorophyll at the mouth of Arakawa river (collected from^36^). (**b**) Monthly average primary productivity of phytoplankton at the mouth of Arakawa river (collected from^36^). (**c**) Overall seasonal variation in the concentration of chlorophyll at region 4 and around region 4^35^.

From Fig. 4a^36^, we observe that chlorophyll remains high at that depth (10 m). One of the dominant phytoplankton species at the mouth of Arakawa river in early summer season (May–June) is Diatom *Skeletonema Costatum*^35^, which grows very well at high temperature (like $23\,^{\circ }$C), also its productivity increases due to salinity of (20–32) ${\text {PSU}}$^37^. Due to this, primary productivity also remains very high during that time (Fig. 4b). Most of phytoplankton production is dominated by *Skeletonema Costatum* at this zone^35^. This results in higher value ($2\,{\mathrm{day}}^{-1}$) of *C* in the month of May (since maximum growth of dominant phytoplankton species is possible at that time due to high primary productivity), hence corresponding mortality *D* should remain low. As overall range of *D* is (0.07–0.2) ${\mathrm{day}}^{-1}$ and *C* is high, we calculate (as before) the overall range of $\varepsilon$ to lie in (0.035, 0.1). It should be noted that among all depths of considered sampling stations which have been taken under consideration for this experiment, depth of Region 3, nearly 10 m, is the lowest. Therefore, among all those stations where data was collected in May 2011, productivity should be highest here, which results in higher value of *C* for this region. Hence, values of $\varepsilon$ will be near to 0.035 for this zone.

### Region 4: Inside Tokyo Bay (different location)

Data was collected from a depth of nearly 50 m inside Tokyo Bay (Region 4) during the months of Dec 2006, Sep 2007 and Feb 2008. It is observed that each season represents different kind of phytoplankton distribution at that particular region (Fig. 1d,e,f).

#### Dec, 2006

During the month of December, average daylight and sunshine hours at and around Region 4 are 9.8 h and 5.7 h respectively (Fig. 2a), which are low compared to that of the previous months. Also, yearly observed report provided by weather atlas (Fig. 2b) indicates low solar radiation intensity (UV index $\approx$ 2) in the month of December. Since sea-surface temperature is mostly controlled by these three important factors, it cannot increase significantly. However, due to 108.7 mm rainfall (Fig. 2c^38^) and 0.8 average snowfall days^38^, sea-surface temperature also decreases, which results in low average sea-surface temperature (17.2–$19.5\,^{\circ }$C)^39^. These environmental factors not only control temperature of water but also influence salinity. Region 4 is nearer to the northern part of Pacific ocean, so salinity of this zone is higher than that of Region 3. It is observed that as depth increases, salinity also increases (Table 2). Also, according to the yearly observational data regarding chlorophyll density in and near region 4 between 1997 to 1998, chlorophyll density remains comparatively low during winter season (Dec–Feb). This means maximum growth of phytoplankton is low, productivity of phytoplankton reduces and its density decreases. In such a scenario, without any loss of generality, we consider the mortality rate (*D*) of phytoplankton is high enough so that density of nutrient increases (NP system is closed). Since, at the depth of nearly 50 m of Region 4 in the month of December, *C* was low and *D* was high, range of $\varepsilon$ will be (0.35–1.0) (as shown in case of Region 1).

**Table 2:** Salinity versus depth at Region 4 of Tokyo Bay in Winter and Autumn^34^.

Depth (m)	Winter (PSU)	Autumn (PSU)
3	32.4–33.4	32.8
10	33.0	33.0
15	32.8–33.6	33.2

#### Sep, 2007

During the month of September, average daylight hours and average sunshine hours at Region 4 are respectively 12.4 h and 5.5 h (Fig. 2a), which is good enough to increase the surface temperature despite low solar radiation intensity (UV index 8, Fig. 2b). Though average rainfall of 131.1 mm (Fig. 2c) brings down the sea temperature, but greater sunshine hours and moderate solar radiation intensity provide suitable temperature for the maximum growth of phytoplankton. Also it is observed that, as depth increases, salinity of sea water increases in this region (Table 2). Figure 3d^40^ indicates that density of phytoplankton remains high and phytoplankton species like Diatom *Skeletonema Costatum*^35^, which can endure high salinity, shows maximum growth^37^. Data was collected from the depth of 50 m at Region 4, where temperature is lower that of the sea-surface but salinity is higher. Since, density of phytoplankton is high at the depth of 50 m and temperature is optimal for photosynthesis, the value of *C* is maximum due to these environmental influences. Therefore, we assume that in the month of September 2007, at that depth of Region 4, *C* was very high, due to high productivity of phytoplankton and better environmental factors, mortality of phytoplankton (*D*) was comparatively low during this time. Accordingly $C\approx 2\,{\mathrm{day}}^{-1}$ and *D* ∈ (0.07, 0.2), hence, $\varepsilon \left( =\frac{D}{C}\right)$ ∈ (0.035, 0.1).

#### Feb, 2008

Average daylight and sunshine hours at Region 4 are respectively 10.9 h and 5.9 h (Fig. 2a). Solar radiation (UV index = 3, Fig. 2b) also remains low during that period with minimum rainfall (average rainfall = 58.2 mm, Fig. 2c^38^). However, due to high occurrence of snowfall (average snowfall = 50 mm^38^), sea-temperature remains lowest at Region 4^41^. Table 2 indicates that in the month of Feb, salinity remains average to high at region 4 and increases with the depth. But, owing to low intensity of light and low water temperature affects the productivity, which results in low density of chlorophyll at this zone (Fig. 4c, station O2 and O6), which ultimately results in reducing growth of phytoplankton. Since maximum growth rate of phytoplankton (*C*) is low due to low productivity, *C* decreases as the depth increases, which implies mean phytoplankton $P_0$ also remains low and decreases with increasing depth. Since the NP system is closed, mean of nutrient should be high and increase with depth, therefore mortality rate of phytoplankton (*D*) is high at Region 4 and increases with depth. Since *D* is high and *C* is low at the depth of 50 m, range of $\varepsilon$ will be same (0.35, 1) as estimated in Region 1 ($\approx 200\,{\mathrm{m}}$, May, 2011) and in Region 4 ($\approx 50\,{\mathrm{m}}$, Dec, 2006). However, value of $\varepsilon$ should be low to medium (not near 1) as the season is Spring and the depth is comparatively low.

## Numerical results

From Fig. 1 we observe that the coefficient of variation varies from less than 1 to greater than 1. The mean and standard deviation are equal along the diagonal line, indicating that the coefficient of variation is equal to 1 along this line. The coefficient of variation varies on both sides of the diagonal depending on the value of the parameter $\beta$. As $\beta$ increases, the coefficient of variation increases. The change of $\beta$ $\left( =\frac{B}{A^2}\right)$ for a fixed region implies the change of the fluctuating components of the system (since *A* is constant), which is characterized by *B*, the variance of overall fluctuating components. Hence, as *B* increases, the coefficient of variation increases.

**Figure 5:**
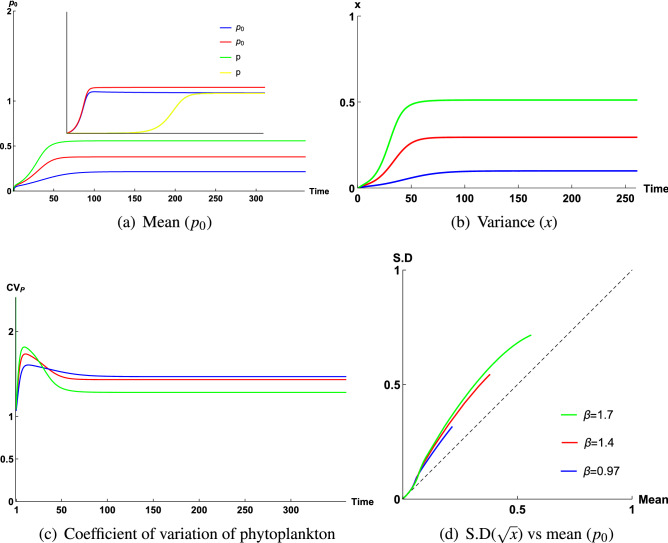
Time series graph of (**a**) mean ($p_0$), (**b**) variance (*x*), (**c**) coefficient of variation of phytoplankton ($CV_P$) and (**d**) corresponding parametric plot of coefficient of variation of phytoplankton ($CV_P$) of the closure model, when only $\beta$ varies for a fixed $\varepsilon$, where $\varepsilon \in (0.35, 1)$, considering total biomass is high, that is, A = 2 $\upmu \,{\mathrm{g}}\,{\mathrm{N}}\,{\mathrm{l}}^{-1}$. The constant parameter values for this simulation are $\kappa = 0.5$ ($K=1\,\upmu \,{\mathrm{g}}\,{\mathrm{N}}\,{\mathrm{l}}^{-1}$, $A=2\,\upmu \,{\mathrm{g}}\,{\mathrm{N}}\,{\mathrm{l}}^{-1}$) and $\varepsilon = 0.95$ for $\beta =0.97, 1.4, 1.7$ respectively.

The closure system is now investigated numerically with the estimated parameter values given in Table 3. Figure 5a,b,c show the time variation of variables $p_0$, *x*, $CV_P$, which correspond to mean, variance and coefficient of variation of phytoplankton respectively at Region 1, considering the total biomass of that zone is high ($A=2\,\upmu \,{\mathrm{g}}\,{\mathrm{N}}\,{\mathrm{l}}^{-1}$). The values of $\kappa$ and $\varepsilon$ are kept constant in these plots ($\kappa =0.5,\,\varepsilon =0.95$). As $\beta$ increases, phytoplankton mean and variance increase (Fig. 5a,b) whereas $CV_P$ of phytoplankton decreases and is greater than 1 (Fig. 5c). The normalized mean $\left( \frac{P_0}{A}\right)$ and standard deviation $\left( \frac{\sqrt{P^{\prime 2}}}{A}\right)$ corresponding to phytoplankton density for different values of $\beta$ are shown in Fig. 5d. Each point in this plot represents the steady state value of mean and standard deviation at different values of $\varepsilon$ $\left( =\frac{D}{C}\right) .$ It is evident that the value of $\varepsilon$ increases with depth implying that the maximum growth rate of phytoplankton decreases due to the reduction of light. Therefore, for a particular value of $\varepsilon$, which means at a particular depth, depending on the variation of total variance (*B*) of the system, $\beta$ is varied to see how fluctuation of overall spread (*B*) of the system affect phytoplankton distribution. It is observed that as $\beta$ increases, the overall spread also increases (both phytoplankton and nutrient). This dynamics is in good agreement with the field observation of phytoplankton data (Fig. 2a).

**Table 3:** Definition of different quantities (parameters) used in the model, their dimensions and estimated values.

Quantity	Definition	Dimension	Estimated parameter values	Scaling factor	Dimensionless quantity	Corresponding dimensionless value
*A*	Sum of nitrate	$\upmu \,{\mathrm{g}}\,{\mathrm{N}}\,{\mathrm{l}}^{-1}$	1.5 or 2.0^42^	–	–	–
*B*	Variance of sum of fluctuating components	$(\upmu \,{\mathrm{g}}\,{\mathrm{N}}\,{\mathrm{l}}^{-1})^2$	–	$\frac{B}{A^2}$	$\beta$	–
*C*	Maximum growth rate of phytoplankton	${\mathrm{day}}^{-1}$	0.2 or 2^22^	–	–	–
*K*	Nutrient uptake half-saturation constant	$\upmu \,{\mathrm{g}}\,{\mathrm{N}}\,{\mathrm{l}}^{-1}$	0.6–1.8^23^	$\frac{K}{A}$	$\kappa$	0.5–4
*D*	Phytoplankton death rate	${\mathrm{day}}^{-1}$	0.07–0.2^22^	$\frac{D}{C}$	$\varepsilon$	0.035–0.1 (for *C* = 2 ${\mathrm{day}}^{-1}$); 0.35–1 (for *C* = 0.2 ${\mathrm{day}}^{-1}$)
$P_0$	Mean phytoplankton	$\upmu \,{\mathrm{g}}\,{\mathrm{N}}\,{\mathrm{l}}^{-1}$	–	$\frac{P_0}{A}$	$p_0$	–
$N_0$	Mean nutrient	$\upmu \,{\mathrm{g}}\,{\mathrm{N}}\,{\mathrm{l}}^{-1}$	–	$\frac{N_0}{A}$	$n_0$	–
$\langle P^{\prime 2}\rangle$	Variance for phytoplankton	$\left( \upmu \,{\mathrm{g}}\,{\mathrm{N}}\,{\mathrm{l}}^{-1}\right) ^2$	–	$\langle (\frac{P^{\prime}}{A})^2\rangle$	*x*	–
$\langle N^{\prime 2}\rangle$	Variance for nutrient	$\left( \upmu \,{\mathrm{g}}\,{\mathrm{N}}\,{\mathrm{l}}^{-1}\right) ^2$	–	$\langle (\frac{N^{\prime}}{A})^2\rangle$	*y*	–
$\langle N^{\prime}P^{\prime}\rangle$	Covariance	$\left( \upmu {\mathrm{g}}\,{\mathrm{N}}\,{\mathrm{l}}^{-1}\right) ^2$	–	$\frac{\langle N^{\prime}P^{\prime}\rangle }{A^2}$	*z*	–
t	Time	day	–	*tC*	$\tau$	–

Figure 5a (inset) shows the time series graph corresponding to the phytoplankton variable for both closure and non-closure model. The use of closure model is evident from the limit of the parameter $\beta$ where the steady state of the closure model coincides with the steady state of the non-closure model (Fig. 5a (inset)). However, the steady state levels do not coincide as $\beta$ increases beyond the critical value $\beta =\beta ^{*}=0.350171$ (for $\kappa =0.5, \varepsilon =0.4$). It is also observed that the value of $\beta ^{*}$ decreases with the increase of the values of $\varepsilon$, which means as the phytoplankton mortality rate (*D*) decreases or as the maximum growth of phytoplankton (*C*) increases, the critical value of $\beta$ increases. Similarly, as the value of $\kappa$ $\left( =\frac{K}{A}\right)$ decreases, $\beta ^{*}$ increases. In a similar manner, the dynamics of mean, variance and $CV_P$ of phytoplankton for fixed $\kappa , \beta$ ($\kappa =0.5, \beta =1.2$) and varying $\varepsilon$ ($\varepsilon =0.8, 0.9, 0.98$) can be shown (see Supplementary Fig. S1). As $\varepsilon$ increases, phytoplankton mean and variance decrease (Fig. S1a, Fig. S1b) whereas $CV_P$ of phytoplankton increases and is greater than 1 (Fig. S1c, Fig. S1d). Therefore, for a particular value of $\beta$, the depth profile is obtained for different $\varepsilon$ values. From these figures, we observe that the mean values of phytoplankton and its standard deviation decrease with the increase in depth, implying that the mean of phytoplankton and its spatial variability decrease with depth. This is a good match with the field observation of phytoplankton data (Fig. 2a).

**Figure 6:**
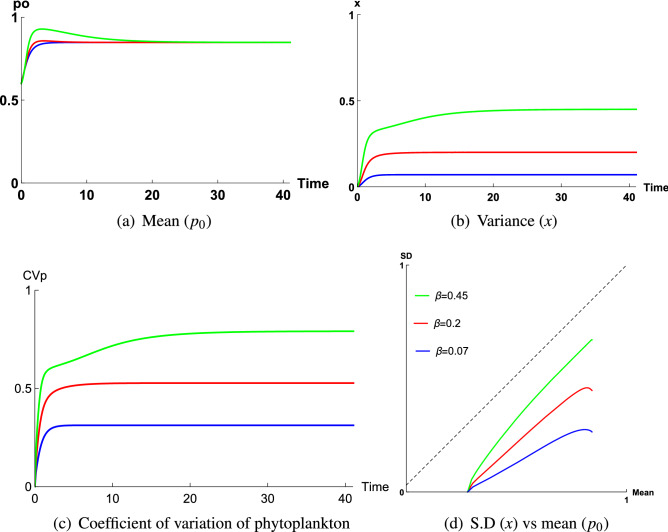
Time series graph of (**a**) mean ($p_0$), (**b**) variance (*x*), (**c**) coefficient of variation of phytoplankton ($CV_P$), and (**d**) corresponding parametric plot of coefficient of variation of phytoplankton ($CV_P$) of the closure model, when $\beta$ varies for a fixed $\varepsilon$, where $\varepsilon \in (0.035, 0.1)$, considering total biomass is high, that is, A = 2 $\upmu \,{\mathrm{g}}\,{\mathrm{N}}\,{\mathrm{l}}^{-1}$. The constant parameter values for this simulation are $\kappa = 0.5$ ($K=1\,\upmu \, {\mathrm{g}}\, {\mathrm{N}}\,{\mathrm{l}}^{-1}$, $A=2\,\upmu \,{\mathrm{g}}\,{\mathrm{N}}\,{\mathrm{l}}^{-1}$) and $\varepsilon =0.085$ for $\beta =0.07, 0.2, 0.45$ respectively.

Figure 6 gives the time series graphs of mean ($p_0$), variance (*x*), coefficient of variation of phytoplankton ($CV_P$), along with a parametric plot in Region 2 (inside Tokyo Bay), Region 4 (inside Tokyo Bay, different location) and Region 3 (mouth of Arakawa river) respectively. The values of $\kappa$ and $\varepsilon$ are kept constant ($\kappa =0.5, \varepsilon =0.085$) and $\beta =0.07, 0.2, 0.45$. in these plots. Figure 6a,b indicate that *x* increases with increasing $\beta$, whereas $p_0$ does not increase noticeably for chosen $\beta$ values. But for $\kappa =0.5$, $\varepsilon =0.085$, $\beta$ can vary up to 0.7 and if we choose $\beta =0.45$, 0.7, then increment rate of $p_0$ will be slightly high than this. However, $CV_P$ also increases with $\beta$ (Fig. 6c) and always remains less than 1 for all $\beta$ values ($\beta \le 0.7$ for $\kappa =0.5$, $\varepsilon =0.085$), which is also confirmed from the parametric plot (Fig. 6d). Again, the dynamics of mean, variance and $CV_P$ of phytoplankton for fixed $\kappa , \beta$ ($\kappa =0.5$, $\beta =0.3$) and varying $\varepsilon$ ($\varepsilon =0.037, 0.06, 0.099$) can also be obtained (see Supplementary Fig. S2). As $\varepsilon$ increases, phytoplankton mean decreases, whereas variance (*x*) does not vary with $\varepsilon$ for a fixed $\beta$ (Fig. S2a, Fig. S2b). But $CV_P$ of phytoplankton increases (Fig. S2c) and is less than 1. Now, for Region 2, $\varepsilon$ can vary from 0 to 0.1. Here, we have varied $\varepsilon$ from low to high for fixed $\beta =0.4$, $\kappa =0.5$, thereafter, we have seen that, time series graph and corresponding parametric plot (Fig. S2d) of $CV_P$ always remain less than 1 for all values of $\varepsilon$. Since $\varepsilon$ can reach up to 0.1, it is never possible to have a value of $\varepsilon$ for which $CV_P$ will be greater than 1.

**Figure 7:**
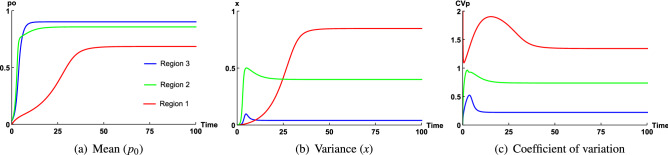
Time series graph of (**a**) mean ($p_0$), (**b**) variance (*x*), (**c**) coefficient of variation of phytoplankton ($CV_P$) of the closure model at Region 2, Region 3 and Region 1 in May 2011. The constant parameter values for this simulation are $\kappa = 0.3$ ($K=0.6\,\upmu \,{\mathrm{g}}\,{\mathrm{N}}\,{\mathrm{l}}^{-1}$, $A=2\,\upmu \,{\mathrm{g}}\,{\mathrm{N}}\,{\mathrm{l}}^{-1}$) for Region 1 and $\kappa = 0.5$ ($K=1\,\upmu \,{\mathrm{g}}\,{\mathrm{N}}\,{\mathrm{l}}^{-1}$, $A=2\,\upmu \,{\mathrm{g}}\,{\mathrm{N}}\,{\mathrm{l}}^{-1}$) for Region 2, Region 3 and $\beta =1.6, 0.04, 0.4$ for $\varepsilon =0.93, 0.037, 0.075$ corresponding to Region 1, Region 3 and Region 2 respectively.

Figure 7 represents the time variation of variables $p_0, x, CV_P$ of closure model at Region 1, Region 2 and Region 3 in May 2011. These figures show how at a particular season, only regional variation and differences in depth affect dynamics of $p_0, x$, which causes $CV_P$ to be greater than 1 at Region 1 and less than 1 in Region 2, Region 3.

**Figure 8:**
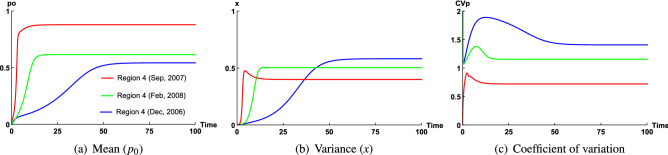
Time series graph of (**a**) mean ($p_0$), (**b**) variance (*x*), (**c**) coefficient of variation of phytoplankton ($CV_P$) of the closure model for Region 4 in Sep 2007, Dec 2006 and Feb 2008 respectively. These figures represent how seasonal variation affect $CV_P$ at the depth of 50 m of Region 4 (inside Tokyo Bay), where range of $\varepsilon$ remains (0.035, 0.1) when productivity is high, and (0.35, 1) when productivity is low, considering total biomass $A=1.5\,\upmu \,{\mathrm{g}}\,{\mathrm{N}}\,{\mathrm{l}}^{-1}$ is low at Region 4 in Sep 2007 and total biomass $A=2\,\upmu \,{\mathrm{g}}\,{\mathrm{N}}\,{\mathrm{l}}^{-1}$ is high at Region 4 in winter season, Dec 2006 and Feb 2008. The constant parameter values for this simulation are $\kappa = 0.4$ ($K=0.6 \,\upmu \,{\mathrm{g}}\,{\mathrm{N}}\,{\mathrm{l}}^{-1}$, $A=1.5\, \upmu {\mathrm{g}}\,{\mathrm{N}}\,{\mathrm{l}}^{-1}$) in Sep 2007 and $\kappa = 0.4$ (K = 0.8 $\upmu \,{\mathrm{g}}\,{\mathrm{N}}\,{\mathrm{l}}^{-1}$, $A=2\,\upmu \,{\mathrm{g}}\,{\mathrm{N}}\,{\mathrm{l}}^{-1}$) in Dec 2006 and Feb 2008 and $\beta =0.4, 0.9, 1.6$ for $\varepsilon =0.085, 0.75, 0.95$ in Sep, Feb and Dec respectively.

Figure 8 represents the time variation of variables $p_0$, *x*, $CV_P$ at Region 4 in Sep 2007, Feb 2008 and Dec 2006. These figures show how seasonal variation affect $CV_P$ at the depth of 50 m of Region 4 (inside Tokyo Bay), where range of $\varepsilon$ remains (0.035, 0.1) when productivity is high, and (0.35, 1) when productivity is low, considering total biomass to be comparatively low at this zone ($A=1.5\,\upmu \,{\mathrm{g}}\,{\mathrm{N}}\,{\mathrm{l}}^{-1}$) in Sep 2007 and total biomass remains high ($A=2\,\upmu \,{\mathrm{g}}\,{\mathrm{N}}\,{\mathrm{l}}^{-1}$) in Dec 2006 and Feb 2008. As total biomass decreases, half-saturation constant will also decrease. We have considered $K=0.8\,\upmu \,{\mathrm{g}}\,{\mathrm{N}}\,{\mathrm{l}}^{-1}$ for this zone in Dec 2006 and Feb 2008, since *A* is low in Sep 2006, therefore, we have considered $K=0.6\,\upmu \,{\mathrm{g}}\,{\mathrm{N}}\,{\mathrm{l}}^{-1}$ for this region in Sep 2007. In Fig. 8a, we observe that $p_0$ remains higher in Sep, whereas $p_0$ remains low in Dec, Feb. For larger domain of $\varepsilon$, range of $\beta$ increases when productivity is low and it remains low when productivity is high. Our numerical outcome resonates with field observation when total biomass remains slightly low at Region 4 in Sep 2007 and it remains high like all other regions at Region 4 in Dec 2006 and Feb 2008.

The time variation of variables $p_0$, *x*, $CV_P$ at Region 4 and Region 1 in Feb 2008, Dec 2006 and May 2011 respectively can also be obtained (see Supplementary Fig. S3). These figures show how seasonal and regional variations affect $CV_P$ at the depth of 50 m of Region 4 (inside Tokyo Bay, only seasonal variation) and at the depth of 200 m of Region 1 (mouth of Tokyo Bay, both seasonal and regional variation), where range of $\varepsilon$ remains (0.35, 1), considering total biomass to be high at all regions ($A=2\,\upmu \,{\mathrm{g}}\,{\mathrm{N}}\,{\mathrm{l}}^{-1}$), only half-saturation constant $K=0.6\,\upmu \,{\mathrm{g}}\,{\mathrm{N}}\,{\mathrm{l}}^{-1}$ for Region 1, whereas $K=0.8\,\upmu \,{\mathrm{g}}\,{\mathrm{N}}\,{\mathrm{l}}^{-1}$ for Region 4 in Dec, Feb. Numerical outcome supports experimental observation under such values of parameters *A*, *K*. We observe that $p_0$ remains higher for Region 1 in May 2011, whereas $p_0$ remains low for Region 4 in Dec 2006, Feb 2008 and $p_0$ is slightly higher in Feb than Dec at Region 4 (Fig. S3a). Also, spread of phytoplankton is higher for Region 1 in May 2011 and it is comparatively low for Region 4 in Dec 2006 and Feb 2008, though in Dec 2006, spread is slightly higher at Region 4 (Fig. S3b). Finally, time series graph of $CV_P$ of Region 1 is lower than that of Region 4 in December but higher than that of Region 4 in February (Fig. S3c).

The variation of variables $p_0$, *x*, $CV_P$ at Region 2, Region 3 and Region 4 in May 2011, Sep 2007 and May 2011 respectively, was obtained (see Supplementary Fig. S4) considering total biomass to be high ($A=2\,\upmu \,{\mathrm{g}}\,{\mathrm{N}}\,{\mathrm{l}}^{-1}$) at Region 2, Region 3 in May 2011 and total biomass is slightly low ($A=1.5 \,\upmu \,{\mathrm{g}}\,{\mathrm{N}}\,{\mathrm{l}}^{-1}$) at Region 4 in Sep 2007, half-saturation constant $K=0.6\,\upmu \,{\mathrm{g}}\,{\mathrm{N}}\,{\mathrm{l}}^{-1}$ for Region 4, whereas $K=1 \,\upmu \,{\mathrm{g}}\,{\mathrm{N}}\,{\mathrm{l}}^{-1}$ for Region 2, Region 3. Numerical outcome supports experimental observation under such values of parameters *A*, *K*. We observe that $p_0$ remains higher for Region 3 in May 2011, $p_0$ remains low at Region 2 and $p_0$ is slightly higher at Region 4 but it is low compared to Region 3 (Fig. S4a). Also, spread (*x* ) of phytoplankton is higher for Region 2, Region 3 but it is very low for Region 3 (Fig. S4b). Finally, time series graph of $CV_P$ of Region 4 is lower than that of Region 2 but higher than that of Region 3 (Fig. S4c).

## Discussion

The coefficient of variation of phytoplankton ($CV_P$) varies with the changes in environmental factors, namely, light, temperature and salinity and many more. The focus of our discussion will be on the variation of $CV_P$ of phytoplankton.

### Case 1: $CV_P < 1$

Measured $CV_P$ values are 0.32, 0.37, 0.78 at the depth of 10 m, 50 m, 50 m of Region 3, Region 4 and Region 2 respectively. From Fig. 1c, we observe that for Region 3, concentrated mean of phytoplankton has escalated over a larger domain along the horizontal axis, while spread of phytoplankton is comparatively very low and constant for all times, whereas for Region 2 and Region 4 (Fig. 1,b,e), spread of phytoplankton is comparatively high, but, quantity of concentrated biomass is higher at Region 4 than Region 2, which is also supported by higher phytoplankton productivity at Region 4 than Region 2.

Nature of spread of phytoplankton is obtained from the dynamics of normalized variance *x* of phytoplankton, which depends on $\beta$. At a fixed depth, *x* increases with increasing $\beta$ (Fig. 5b). For all regions where $CV_P<1$, domain of $\varepsilon$ belongs to (0.035, 0.1), $\beta$ is very sensitive to half-saturation constant *K* and total biomass *A* of the system. If *A* decreases, then corresponding *K* will decrease, in this case, range of domain of $\beta$ reduces. Again, for fixed biomass, if *K* increases, then range of $\beta$ decreases.

We already know, $\varepsilon$ value remains higher for Region 2 and Region 4, whereas for Region 3, it is very low. Hence for fixed *A*, *K*, domain of $\beta$ will be larger for Region 2, Region 4 compared to that of Region 3. So, $\beta$ value will be very low for Region 3 and therefore overall spread (*x*) of dominating phytoplankton community remains very low at this zone during observatory period. But, on the other hand highest phytoplankton productivity among all other regions causes phytoplankton biomass to dominate most of the total biomass of the system and hence $p_0$ remains close to 1 (Fig. 7a). This nature of spread and mean of phytoplankton has also been observed in field observation (Fig. 1c). From Fig. 1c, we observe that mean of phytoplankton spreads up to 30 units, mostly concentrated between 2 and 20 units. This density of phytoplankton mean is highest among all other regions, whereas spread of phytoplankton is very low. Therefore, since $\beta$ is the lowest depending on lowest value of $\varepsilon$ caused by highest productivity at Region 3 in May 2011, $p_0$ remains close to 1 (Fig. 7a) and *x* is very low (Fig. 7b), hence, this fact causes $CV_P$ to be the lowest ($CV_p=0.32$) at this zone (Fig. 7c). Similar dynamics is also observed for Region 2 in May 2011 and Region 4 in Sep 2007 with phytoplankton biomass ($p_0$) is substantially less due to low productivity compared to the mouth of Arakawa river in May, 2011 (Region 3).

In case of Region 2 and Region 4, $\varepsilon$ values are high (slightly higher in Region 4), hence, domain of $\beta$ will be slightly higher for Region 4 compared to Region 2. Now, if $\beta$ increases, spread *x* will also increase. But, from Fig. 1e, we observe that spread of phytoplankton is concentrated between 1 and 1.7 units, which implies $\beta$ to be low for this region. Now, domain of $\beta$ decreases depending on two facts, (i) either total biomass *A* of the system was low (ii) for fixed total biomass $A=2$, half-saturation constant *K* was high at this zone in Sep 2007. If *K* would be high, then phytoplankton density would also be very high. But from Fig. 1e, we can observe that phytoplankton biomass has only spread up to 3 units along *x*-axis, it is mostly concentrated between 0.5 and 2 units. So, the second fact is not valid for this zone. Instead, if we consider that total biomass was low at this zone, then phytoplankton biomass will also be low, which explains the nature of mean in Fig. 1e. Therefore, for low total biomass, range of $\beta$ will be very low, which indicates the spread of phytoplankton is also very low. Now, in Sep, productivity remains very high at this zone, therefore, though total biomass is low but most of the system biomass will be dominated by phytoplankton biomass and hence $p_0$ will be close to 1 (Fig. 8a), whereas for very low $\beta$ value, spread of phytoplankton will be very low compared to this value of $p_0$ (Fig. 8b), which generates $CV_P=0.35$ (Fig. 1e).

In Region 2, mean and spread of phytoplankton can reach up to 2.5 and 2 units respectively (Fig. 1b) and measured $CV_P=0.78$, which is the highest. Though productivity remains high in Region 2 in May 2011 but compared to Region 3, it is very low. As a result, distribution of mean ($p_0$) is low in Region 2, where this distribution is high in Region 3 (Fig. S4a), as a result, $\varepsilon$ values remain high for Region 2 than Region 3 (for numerical results, chosen $\varepsilon =0.037$ for Region 3 while $\varepsilon =0.075$ for Region 2). Therefore domain of $\beta$ increases for Region 2 than Region 3 (since high $\varepsilon$ values generate high $\beta$). On the other hand, $\varepsilon$ values remain slightly low for Region 2 than Region 4, due to slightly higher productivity at Region 4 in Sep 2007. Hence, phytoplankton biomass ($p_0$) remains low for Region 2 compared to Region 4 (Fig. S4a), which has also been observed in field observation (Fig. 1b), where concentrated phytoplankton biomass is very low (dense around 0.5 units) for Region 2 than Region 4. So, for higher range of $\varepsilon$, domain of $\beta$ should remain higher for Region 4 than Region 2. But, since Region 4 belongs to a zone where total biomass (*A*) is low, range of $\beta$ remains more or less the same for both regions, which indicates spread of phytoplankton remains nearly the same for both regions (Fig. S4b). But, $p_0$ remains low for Region 2 than Region 4 (Fig. S4a), which causes $CV_P$ to be higher for Region 2 than Region 4 (Fig. S4c). Again, though $p_0$ remains comparatively low for Region 2 than Region 4, still due to higher productivity, most of the total biomass (*A*) is dominated by phytoplankton biomass. As a result, $p_0$ remains close to 1 (Fig. 7a), whereas due to overall low range (0.035, 0.1) of $\varepsilon$ caused by high productivity, range of $\beta$ remains low compared to the range of $\beta$ corresponding to those zones where $CV_P>1$. Therefore, spread *x* remains comparatively low (Fig. 7b), whereas $p_0$ is close to 1 (Fig. 7a), which causes $CV_P$ to be less than 1 (Fig. 7c) at this zone.

From above discussion we observe that when $\varepsilon$ belongs to (0.035, 0.1) and due to this range of $\varepsilon$, domain of $\beta$ reduces for a location, then $CV_P$ remains less than 1 at that zone. These domains of $\varepsilon , \beta$ are determined from nature of phytoplankton productivity at a location during the period of observation and nature of the spread of dominating class. It has been observed that in case of Region 3, during early summer season (May), the existing phytoplankton communities are *Skeletonema Costatum*, *Navicula species* and *Pyraminonas Grossii*^36^, for Region 4, the existing phytoplankton communities in Sep are diatom *Skeletonema Costatum*, Dinoflagellates, Raphidophytes and others^35^, whereas for Region 2, the existing classes in May are diatom *Skeletonema Costatum*, Raphidophytes and others^35^. But, for all three regions during corresponding time periods, most of the phytoplankton biomass is dominated by the diatom class, *Skeletonema Costatum*^35,36^. Spread of this phytoplankton class has a peculiar nature, which is influenced by its measure of stickiness $\alpha$, where $\alpha \in (0,\,0.98)$^43^. Now, during the period of observation, since the dominating class *Skeletonema Costatum* coexists with some other phytoplankton classes at all three regions, therefore range of its measure of stickiness $\alpha$ should belong to (0.02, 0.25) for these regions and depending on $\alpha$, scatteredness of *Skeletonema Costatum* has varied for these zones, that is, when $\alpha$ is high, scatteredness of *Skeletonema Costatum* reduces and when $\alpha$ is low, this scatteredness increases. In field observation, we have seen that, at Region 3, scatteredness of *Skeletonema Costatum* is very low in May 2011, whereas for Region 4 and Region 2, it is slightly higher in Sep 2007 and May 2011. For all three zones, $\alpha$ belongs to $(0.02,\,0.25)$ but its value has varied differently for each zone. If we consider $\alpha$ to be high for Region 3 in May 2011, then *Skeletonema Costatum* will be more sticky for that zone during that time period which will hinder the scatteredness. If we assume $\alpha$ to be slightly high for Region 2, Region 4 for corresponding time periods, then *Skeletonema Costatum* will be less sticky than Region 3 and scatteredness will be slightly higher for these zones by that time.

In the model, spread due to scatteredness is controlled by low $\beta$ value. Therefore, ecologically it might be considered that during early summer at Region 3, $\alpha$ value was close to 0.25, which has caused *Skeletonema Costatum* to remain more sticky at that zone, as a result, spread was very low which represents low $\beta$ value. Similar ecological assumptions can be drawn in case of Region 2, Region 4, but the only difference is probably, for these two zones in summer and early spring season respectively, $\alpha$ was slightly low than Region 3. As a result, the dominating class *Skeletonema Costatum* was less sticky than Region 3 and spread due to scatteredness was slightly higher than Region 3 (Fig. S4b). Hence, differences in the nature of total biomass of a system, nature of productivity and finally nature of stickiness of dominating phytoplankton species cause high irregularity in phytoplankton distribution and produce low $CV_P$ values for Region 2, Region 3 (Fig. 7c, Fig. S4c) and Region 4 (Fig. 8c, Fig. S4c).

### Case 2: $CV_P > 1$

In case of Region 4, at the depth of 50 m, $CV_P$ remains 1.61 and 1.36 in Dec 2006 and Feb 2008 respectively. In Dec 2006, Feb 2008, due to very low productivity, range of $\varepsilon$ remains (0.35, 1.0) at Region 4, which generates larger domain of $\beta$ (considering total biomass and half saturation constant remain the same at Region 4 during both time periods Dec 2006 and Feb 2008). Since total biomass *A* is conserved, large value of $\beta$ indicates larger value of *B*, which ecologically implies spread of all fluctuating components of nutrient and phytoplankton remains higher. Therefore, in Dec 2006 and Feb 2008, spread of phytoplankton remains higher, whereas due to very low productivity, most of the total biomass *A* is dominated by nutrient biomass $n_0$ and phytoplankton biomass $p_0$ remains very low, that is, $p_0<<1$ (Fig. S1a), which is also observed in field observation, phytoplankton biomass is concentrated between 0.3 and 1.5 units in Dec 2006 (Fig. 1d) and in Feb 2008, it is concentrated between 0.8 and 2 units (Fig. 1f), whereas for both cases, spread of phytoplankton is concentrated between 0.5 and nearly 3 units (Fig. 1d,f), which is higher than $p_0$. Therefore, our numerical result validates the field observation at this zone.

Due to low primary productivity and higher spread of phytoplankton caused by high $\beta$ value causes $CV_P$ to be greater than 1 at this zone in Dec 2006 and Feb 2008. But, due to slightly higher productivity, values of $\varepsilon$ remain slightly low in Feb than Dec, which causes $\beta$ to be slightly high in Dec than Feb, as a result, $p_0$ is slightly higher in Feb than Dec (Fig. 8a) and observed spread of phytoplankton is slightly high in Dec than Feb (because of higher $\beta$ value in Dec) (Fig. 8b), which causes $CV_P$ to be slightly higher in Dec than Feb, $CV_{P\,({\text {in}}\,{\text{Dec}})}=1.61>CV_{P\,({\text {in}}\,{\text{Feb}})}=1.36$ (Fig. 8c). As discussed before, $\beta$ represents the spread of dominating phytoplankton species, which is indirectly related to the nature of stickiness of dominating phytoplankton community. Since $\beta$ remains high at Region 4, higher spread of dominating class *S. Costatum* (less sticky) in Dec and Feb is observed from high resolution data, therefore, in Dec and Feb, measure of stickiness of *S. Costatum* was low at Region 4, which causes higher spread of this class at Region 4, which corresponds to higher $\beta$ value.

At the experimental depth 200 m of Region 1, phytoplankton biomass remains very low due to very low productivity. Therefore, most of the total biomass of this zone is nutrient biomass ($n_0$) and $p_0<<1$, (Fig. 7a), which is also observed in field observation (Fig. 1a). Phytoplankton biomass spreads up to 4.5 units, which happens due to low productivity. Now, because of very low productivity, $\varepsilon$ belong to the range (0.35, 1) and $\varepsilon$ values remain close to 1 due to higher depth. High values of $\varepsilon$ generate larger domain of $\beta$ for this zone, as a result of which spread should also be higher at Region 1 (Fig. 5b), which is actually observed in field observation (Fig. 1a), where S.D is scattered and it has spread up to 9.7 units along vertical axis. Since spread (*x*) remains very high and $p_0$ remains very low, this fact causes $CV_P$ to be greater than 1 at this zone (Fig. 7c).

Since spread of phytoplankton is very high and S.D. of phytoplankton is highly scattered at this zone, this corresponds to the fact that domain of $\beta$ will be larger for this zone than any other zone. Range of $\beta$ increases if (i) both *A*, *K* decrease or (ii) for fixed $A=2\,\upmu \,{\mathrm{g}}\,{\mathrm{N}}\,{\mathrm{l}}^{-1}$, *K* decreases. Since, most of total biomass at the depth of 200 m of Region 1 is dominated by nutrient, phytoplankton is biomass very low. Therefore, it is ecologically meaningful for only *K* to be low for fixed $A (=\,2\,\upmu \,{\mathrm{g}}\,{\mathrm{N}}\,{\mathrm{l}}^{-1})$ at this zone. If we consider half-saturation constant *K* to be low for this zone, then domain of $\beta$ increases, which explains higher spread of phytoplankton at this zone.

Ecologically, higher spread of phytoplankton at this zone can be related to less sticky nature of dominating phytoplankton community. Probably during the period of measurement in May 2011, value of measure $\alpha$ of stickiness of the dominating phytoplankton class was the lowest among all other regions, as a result, dominating class was scattered and has spread most than any other zones. Now, due to seasonal impact (summer season), productivity at this zone was also higher than Region 4 in winter season (Dec 2006, Feb 2008) (Fig. S3a), as a result, $\varepsilon$ values remain higher for Region 4 in Dec than Region 1 in May, but due to low value of *K*, $\beta$ values remain higher for Region 1 in May than any other region, this higher $\beta$ value indicates higher spread of phytoplankton for Region 1 than Region 4 (Fig. S3b). Now, higher $p_0$ has caused $CV_P$ of Region 1 to be less than that of Region 4 in Dec, $CV_{P\,({\text {in}}\,{\text{ Dec}}\,{\text{ at}}\,{\text{ Region}}\,4)}=1.61>CV_{P\,({\text {in}}\,{\text{ May}}\,{\text{ at}}\,{\text{ Region}}\,1)}=1.5$ (Fig. S3c), since productivity at Region 4 is the lowest in Dec. But, in case of Region 4 in Feb, productivity is slightly high, so $\varepsilon$ values are less than that of Region 1 and hence, corresponding domain of $\beta$ is also small, which indicates less spreading of phytoplankton community at Region 4 in Feb (Fig. S3b). Thus, since $p_0$ is comparatively high in Feb than Dec at Region 4 and spread is low, hence, $CV_P\,{\text {in}}\,{\text{ Feb}}\,{\text{ at}}\,{\text{ Region}}\,4 =1.36<CV_P\,{\text {in}}\,{\text{ May}}\,{\text{ at}}\,{\text{ Region}}\,1=1.5$ (Fig. S3c). This variation in $CV_P$ at Region 1 and Region 4 during summer and winter season respectively, is caused by change in the nature of phytoplankton productivity due to seasonal impact and differences in the nature of stickiness of dominating phytoplankton communities at two different zones.

## Conclusion

In the field observation executed for several months between 2006 and 2011, it has been observed that coefficient of variation of phytoplankton ($CV_P$) can be divided into two classes namely, $CV_P>1$ and $CV_P<1$ (Fig. 1) at different locations of Tokyo Bay during different time periods. The variation of $CV_P$ is interconnected to the variation of depth with the change of location and seasonal variation. In three different locations having different depths during a fixed month May, 2011 (early summer of that year), $CV_P<1$ for Region 2 (inside Tokyo bay, depth $\approx 50$ m), Region 3 (mouth of Arakawa river, depth $\approx 10$ m) but $CV_P>1$ for Region 1 (mouth of Tokyo Bay, depth $\approx$ 200m). Again, nature of $CV_P$ at a fixed depth of a fixed location also changes with seasonal variation, as observed from the collected data. In summer season $CV_P$ is less than 1 ($CV_P=0.35$) whereas, in winter season ($CV_P=1.32$), it is greater than 1 at Region 4 (inside Tokyo bay, depth $\approx 50$ m). Therefore, it is observed that in late summer-early autumn (May–Sep) season, when measurements are done at upper sea-level (depth $\le$ 50m), $CV_P$ remains less than 1, while in winter season (Dec–Feb), $CV_P$ remains greater than 1 on that same level of sea. But, when measurement is done at a deeper level of sea in summer season (Region 1, mouth of Tokyo Bay, depth $\approx 200$ m, May, 2011), $CV_P$ is greater than 1 at that region. This clearly indicates that environmental factors which vary with seasonal changes and depth have a great impact on this variation of $CV_P$.

This change in $CV_P$ is one of the factors for irregularity or intermittency observed in phytoplankton distribution, which is controlled by phytoplankton productivity, death rate and spread of fluctuating component of phytoplankton. $CV_P$ is also influenced by phytoplankton maximum growth (*C*, influenced by light intensity), mortality (*D*) and spread of dominating phytoplankton community. Again, value of *C* depends on rate of photosynthesis, which is controlled by available quantity of basic elements (temperature, salinity etc.) that varies with variation of depth and months. Therefore, it can be concluded that the environmental influences decide the nature of phytoplankton productivity and hence, the values of *C*, *D*, which in the long run affect the nature of $CV_P$. Spread of phytoplankton is connected to $\beta$ ($=\frac{B}{A^2}$), since in our model for any region, *A* remains constant, variation of $\beta$ indicates variation of *B*, that is, variation of total variance of the system. Increment of $\beta$ indicates that *B* increases, which indicates increment of variances of phytoplankton and nutrient, that is, spread of phytoplankton also increases. This $\beta$ varies with the variation of total biomass *A*, half-saturation constant *K* of a region. *K* also depends on total phytoplankton biomass (influenced by phytoplankton productivity, hence *C*). In our analysis, the nature of $CV_P$ depends on the values of dimensionless parameters $\kappa$, $\varepsilon$, $\beta$, which are functionally related to $C,\,D,\,K$ and *A*.

For any region of any ocean, sea, lake of any part of the world, (i) if depending on availability of basic elements highly essential for photosynthesis (temperature, salinity, density of chlorophyll etc.), phytoplankton productivity remains very high at that zone and dominating phytoplankton community behaves to be highly sticky, that is, spread of dominating phytoplankton class remains low, then $CV_P$ will be less than 1 at that zone (Table 4), (ii) if due to unavailability of basic elements required for photosynthesis, productivity remains very low at that zone and dominating class behaves to be less sticky at that location, then $CV_P$ will be greater than 1 at that zone (Table 5). Similar dynamics are observed in sub-Antarctic area of the straits of Magellan ($53\,^{\circ }$S) in the spring of 1997 and summer of 1998^44^ and on surface waters of four sampling stations of Funka Bay (Table 6), Japan between Dec 1995 and March 1997^45^ (see Supplementary Figs. S5).

**Table 4:** $CV_P<1$: nature of phytoplankton productivity and spreading triggered by regional and seasonal impact.

Station	Time	Productivity ($p_0$)	Spread of phytoplankton (*x*, $x<=\beta$))	$\beta$	Possible $CV_P$	Measured $CV_P$
Region 2	May 2011	High	Low	Low	$CV_P<1$	0.78
Region 3	May 2011	High	Low	Low	$CV_P<1$	0.32
Region 4	Sep 2007	High	Low	Low	$CV_P<1$	0.37

**Table 5:** $CV_P>1$: nature of phytoplankton productivity and spreading triggered by regional and seasonal impact.

Station	Time	Productivity ($p_0$)	Spread of phytoplankton (*x*, $x<=\beta$)	$\beta$	Possible $CV_P$	Measured $CV_P$
Region 1	May 2011	Very low	High	High	$CV_P>1$	1.5
Region 4	Dec 2006	Very low	High	High	$CV_P>1$	1.61
Region 4	Feb 2008	Low	High	High	$CV_P>1$	1.36

**Table 6:** Variation of $CV_P$ on surface waters of four different sampling stations of Funka Bay, Japan.

Season	No. of observations	Mean	S.D	$CV_P$
March–Oct (Summer)	9	1.09	0.58	$0.53<1$
March–Oct (Summer)	9	0.53	0.40	$0.75<1$
March–Oct (Summer)	21	0.66	0.44	$0.67<1$
March–Oct (Summer)	21	0.87	0.50	$0.57<1$
March–April (Spring)	31	0.57	0.41	$0.72<1$
Feb–March (Winter)	10	2.71	3.71	$1.37>1$
Feb–March (Winter)	10	0.70	0.91	$1.3>1$

We now check whether the variation in the nature of $CV_P$ at different stations of Tokyo Bay in May 2015 can be validated by the proposed model. In case of Region 1, spread of dominating phytoplankton class remains very high in May, therefore since stations O1, O2 are near Region 1 (Table 7), hence for these stations, spread (*x*) will also be higher. But, at these two stations, temperature remains very cold, nearly $-0.04\,^{\circ }$C at the depth of 100 m, density of chlorophyll remains less than 1 $\upmu \,{\mathrm{g}}\,{\mathrm{N}}\,{\mathrm{l}}^{-1}$, as a result, productivity remains very low. Therefore, according to the conclusion generated from the model, due to higher spread of phytoplankton and low productivity, $CV_P$ should be greater than 1 at these zones. Similarly, for station O3, since it is near Region 4 and Region 1, spread of phytoplankton remains high. But, temperature is very low at this zone (as station O1 and station O3 have similar water properties), which results in low productivity of phytoplankton. As a result $CV_P$ should be greater than 1 at this zone. For station O7, ${\text {temperature}}\approx 20\,^{\circ }$C, ${\text {salinity}}\approx 30\,{\text {PSU}}$, density of chlorophyll was greater than 10 $\upmu \,{\mathrm{g}}\,{\mathrm{N}}\,{\mathrm{l}}^{-1}$ for upper 10 m layer, as a result, productivity should be high at this depth. Stations O5, O7 are near Region 2, hence, for these zones, productivity remains high. On the other hand, these stations are near Region 2, Region 3, where spread of phytoplankton is generally very low for high sticky nature of dominating class. Hence, $CV_P$ should be less than 1 at these stations. For station O4, it is near Region 4, where in summer season, spread of phytoplankton remains low (Region 4: Sep, 2007-autumn, late summer), water is less saline, temperature is high and chlorophyll density is average, so productivity should be average. Since spread remains low, hence according to our calculation, $CV_P$ should be less than 1 at this zone. The nature of $CV_P$ obtained from the proposed model and actual measured $CV_P$ of each zone are compared and put in tabular form (Table 8).

**Table 7:** Geographical locations and period of observations of phytoplankton at different sampling stations.

Station	Profile depth (m)	Date of observation	Surrounding regions
Station O1	120	24/05/2015, time-8:35 a.m.	Region 1 (mouth of Tokyo Bay)
Station O2	110	24/05/2015, time-9:50 a.m.	Region 1 (mouth of Tokyo Bay)
Station O3	22	24/05/2015, time-11:27 a.m.	Region 1 (mouth of Tokyo Bay), Region 4 (inside Tokyo Bay)
Station O4	17	24/05/2015, time-12:51 a.m.	Region 4 (inside Tokyo Bay)
Station O5	23	25/05/2015, time-9:57 a.m.	Region 2 (inside Tokyo Bay)
Station O6	18	25/05/2015, time-11:13 a.m.	Region 2 (inside Tokyo Bay), Region 3 (mouth of Arakawa river)
Station O7	15	25/05/2015, time-13:12 a.m.	Region 2 (inside Tokyo Bay), Region 3 (mouth of Arakawa river)

**Table 8:** Comparison of model predicted $CV_P$ values with measured experimental $CV_P$ values at different stations.

Station	Productivity	Spread of phytoplankton	Possible $CV_P$	Measured $CV_P$
Station O1	Very low	High in May	$CV_P>1$	4.6
Station O2	Very low	High in May	$CV_P>1$	4.6
Station O3	Low	High in May	$CV_P>1$	4.6
Station O4	High	Low in summer	$CV_P<1$	0.8
Station O5	High	Low in May	$CV_P<1$	0.5
Station O6	High	Very low in May	$CV_P<1$	0.4
Station O7	High	Very low in May	$CV_P<1$	0.3

Finally, our results suggest that a microscale ecological study with closure approach of the proposed model succeeded in capturing the dynamics of phytoplankton intermittency in different geographical locations of Japan and in sub-Antarctic zone.

## Supplementary Information

Supplementary Information.
