# Supplementary material for: Effect of productivity and seasonal variation on phytoplankton intermittency in a microscale ecological study using closure approach
Source: Sci Rep. 2022 Apr 8;12:5939. doi: 10.1038/s41598-022-09420-5 (PMC8993848; doi:10.1038/s41598-022-09420-5)
Supplement: Supplementary file 1 — Supplementary Information. [file 41598_2022_9420_MOESM1_ESM.pdf]

Effect of productivity and seasonal variation on phytoplankton intermittency in a microscale ecological study using closure approach (Supplementary Information).

Arpita Mondal[1] **and Sandip Banerjee**[2]

[1,2]Indian Institute of Technology Roorkee, Department of Mathematics, Roorkee 247667, Uttarakhand, India
[*]Corresponding author

ABSTRACT

Derivation of mean, variance and co-variance equations for the closure model is done in this section.

1 Formation of Closure Model

We have considered a simple NP (Nutrient-Phytoplankton) model with Holling type III functional response and linear mortality of phytoplankton to understand the mechanism of phytoplankton distribution at different sampling stations of Tokyo Bay, Japan during the period of field observation between 2006 and 2011.

$$\frac{dP}{dt} = C\frac{N^2P}{K^2+N^2} - DP, \tag{1}$$

$$\frac{dN}{dt} = -C\frac{N^2P}{K^2+N^2} + DP, \tag{2}$$

where,

$$\frac{dP}{dt} + \frac{dN}{dt} = 0 \Rightarrow P+N = \text{Constant} = A(say).$$

In this conventional NP-model, influence of fluctuating components of model variables N, P are overlooked whereas, in case of marine ecosystem, nature of phytoplankton distribution is highly influenced by the role play of fluctuating components P', N', which causes disturbances in the mechanism of phytoplankton distribution, which, in the long run, exerts an influence on the nature of CV_P. Hence, we will apply closure technique to our conventional NP model (12) to get a system of equations which also includes the rate of change of both mean and fluctuating components ($P_0, N_0, < P'^2 >, < N'^2 >, < N'P' >$) of our non-closure model variables N, P. This formation of closure systems requires some assumptions to be satisfied, (i) conventional NP model should be closed (ii) components should satisfy dominated-convergence theorem of random variables. It should be noted that our actual model variables N, P are needed to be considered as functions of both time (t) and space (r) so that they can be decomposed into mean (N_0, P_0) and fluctuating components (N', P'), that is,

$$N(r,t) = N_0(r,t) + N^{'}(r,t), \tag{3}$$

$$P(r,t) = P_0(r,t) + P^{'}(r,t), \tag{4}$$

Here P_0 and N_0 are spatial mean values of P, N respectively, $N^{'}$, $P^{'}$ are their respective fluctuating components corresponding to each mean value. The horizontal and vertical sampling for microscale phytoplankton distribution have the same statistics at the centimeter scale, also at the millimeter scale (except for extreme values). Hence, the statics of the fluctuating components is independent of the direction of sampling (isotropic). Therefore, the spatial average of each fluctuating component is zero at any particular time, that is $< P^{'}(r) >=0$, $< N^{'}(r) >=0$, while its temporal average cannot be zero, which implies $< P(t) >= P_0(t)$ and $< N(t) >= N_0(t)$. With the help of these assumptions we will now formulate closure system of equations.

Using 3, and 4 into 1, we get,

$$\begin{aligned}
\frac{dP}{dt} =& C\frac{N^2P}{K^2+N^2}-DP \\
\Rightarrow \frac{d(P_0+P^{'})}{dt} =& C\frac{(N_0+N^{'})^2(P_0+P^{'})}{K^2+(N_0+N^{'})^2}-D(P_0+P^{'}) \\
\Rightarrow \frac{d(P_0+P^{'})}{dt} =& C\frac{(N_0+N^{'})^2(P_0+P^{'})}{(K^2+N_0{}^2)}\left(1+\frac{N^{'2}+2N_0N^{'}}{(K^2+N_0{}^2)}\right)^{-1}-D(P_0+P^{'})
\end{aligned}$$

(Neglecting third and higher order terms of fluctuating components in the expansion of the series, we obtain,)

$$\begin{aligned}
\Rightarrow \frac{d(P_0+P^{'})}{dt} =& C\frac{(N_0+N^{'})^2(P_0+P^{'})}{(K^2+N_0{}^2)}\left(1-\frac{N^{'2}}{(K^2+N_0{}^2)}-\frac{2N_0N^{'}}{(K^2+N_0{}^2)}+\frac{4N_0{}^2N^{'2}}{(K^2+N_0{}^2)^2}\right)-D(P_0+P^{'}) \\
\Rightarrow \frac{d(P_0+P^{'})}{dt} =& \frac{C(N_0{}^2+N'^2+2N_0N')(P_0+P')}{(K^2+N_0{}^2)}\left(1-\frac{N^{'2}}{(K^2+N_0{}^2)}-\frac{2N_0N^{'}}{(K^2+N_0{}^2)}+\frac{4N_0{}^2N^{'2}}{(K^2+N_0{}^2)^2}\right)-D(P_0+P^{'}) \\
\Rightarrow \frac{d(P_0+P^{'})}{dt} =& \frac{C(N_0{}^2P_0+N'^2P_0+2N_0P_0N'+N_0{}^2P'+N'^2P'+2N_0N'P')}{(K^2+N_0{}^2)}\left(1-\frac{N^{'2}}{(K^2+N_0{}^2)}-\frac{2N_0N^{'}}{(K^2+N_0{}^2)}+\frac{4N_0{}^2N^{'2}}{(K^2+N_0{}^2)^2}\right) \\
&-D(P_0+P^{'}) \\
\Rightarrow \frac{d(P_0+P^{'})}{dt} =& \frac{C}{(K^2+N_0{}^2)}(N_0{}^2P_0+N^{'2}P_0+2N_0P_0N^{'}+N_0{}^2P^{'}+2N_0N^{'}P^{'}) \\
&-\frac{C}{(K^2+N_0{}^2)^2}(N_0{}^2P_0N^{'2}) \\
&-\frac{C}{(K^2+N_0{}^2)^2}(2N_0{}^3P_0N^{'}+4N_0{}^2P_0N^{'2}+2N_0{}^3N^{'}P^{'}) \\
&+\frac{C}{(K^2+N_0{}^2)^3}(4N_0{}^4P_0N^{'2})-D(P_0+P^{'})
\end{aligned}$$

(Neglecting third and higher order terms of fluctuating components we get,)

$$\begin{aligned}
\Rightarrow \frac{d(P_0+P^{'})}{dt} =& \frac{C}{(K^2+N_0{}^2)}(N_0{}^2P_0+N^{'2}P_0+2N_0P_0N^{'}+N_0{}^2P^{'}+2N_0N^{'}P^{'}) \\
&-\frac{C}{(K^2+N_0{}^2)^2}(N_0{}^2P_0N^{'2}+2N_0{}^3P_0N^{'}+4N_0{}^2P_0N^{'2}+2N_0{}^3N^{'}P^{'}) \\
&+\frac{4CN_0{}^4P_0N^{'2}}{(K^2+N_0{}^2)^3}-D(P_0+P^{'})
\end{aligned}$$

After taking average on both sides and using $< P^{'}(r) >=0$, $< N^{'}(r) >=0$, we get,

$$\begin{aligned}
\frac{dP_0}{dt} =& \frac{C}{(K^2+N_0{}^2)}(N_0{}^2P_0+<N'^2>P_0+2N_0<N'P'>)-DP_0 \\
&-\frac{C}{(K^2+N_0{}^2)^2}(N_0{}^2P_0<N'^2>+2N_0{}^3<N'P'>+4N_0{}^2P_0<N'^2>)+\frac{4CN_0{}^4P_0<N'^2>}{(K^2+N_0{}^2)^3} \\
\Rightarrow \frac{dP_0}{dt} =& \frac{CN_0{}^2P_0}{(K^2+N_0{}^2)}+\frac{2CK^2N_0<N'P'>}{(K^2+N_0{}^2)^2}+\frac{CK^2P_0<N'^2>(K^2-3N_0{}^2)}{(K^2+N_0{}^2)^3}-DP_0 \qquad (5)
\end{aligned}$$

Similarly we get,

$$\frac{dN_0}{dt}=-\frac{CN_0{}^2P_0}{(K^2+N_0{}^2)}-\frac{2CK^2N_0<N'P'>}{(K^2+N_0{}^2)^2}-\frac{CK^2P_0<N'^2>(K^2-3N_0{}^2)}{(K^2+N_0{}^2)^3}+DP_0 \qquad (6)$$

Now we have,

$$P(r,t)=P_0(r,t)+P^{'}(r,t)\Rightarrow P'=P-P_0\Rightarrow\frac{dP'}{dt}=\frac{dP}{dt}-\frac{dP_0}{dt}$$
$$\Rightarrow\frac{1}{2}2P'\frac{dP'}{dt}=P'\frac{dP}{dt}-P'\frac{dP_0}{dt}\Rightarrow\frac{1}{2}\frac{d(P'^2)}{dt}=P'\frac{dP}{dt}-P'\frac{dP_0}{dt}$$

(After taking average on both sides, we get,)

$$\Rightarrow<\frac{1}{2}\frac{d(P'^2)}{dt}>=<P'\frac{dP}{dt}-P'\frac{dP_0}{dt}>$$

(Using properties of Reynolds average to exchange the derivative and average on the left side for the random variable $<P'^2>$)

$\left(\text{that is, for any real variable } u,\ <\frac{du}{dt}>=\frac{d<u>}{dt}, \text{ we obtain,}\right)$

$$\Rightarrow\frac{1}{2}\frac{d<P'^2>}{dt}=<P'\frac{dP}{dt}>-<P'\frac{dP_0}{dt}>$$
$$\Rightarrow\frac{1}{2}\frac{d<P'^2>}{dt}=<P'\frac{dP}{dt}>\quad\left(\text{since } <P'\frac{dP_0}{dt}>=0\right)$$

(Now multiplying 1 with P' and using 3 and 4, we obtain,)

$$P^{'}\frac{dP}{dt}=\frac{C}{(K^2+N_0{}^2)}[N_0{}^2P_0P^{'}+N^{'2}P_0P^{'}+2N_0P_0N^{'}P^{'}+N_0{}^2P^{'2}+2N_0{}^2N^{'}P'^2]$$
$$-\frac{C}{(K^2+N_0{}^2)^2}[N_0{}^2P_0N'^2P'+2N_0{}^3P_0N'P'+4N_0{}^2P_0N'^2P'+2N_0{}^3N'P'^2]$$
$$+\frac{4CN_0{}^4P_0N'^2P'}{(K^2+N_0{}^2)^3}-D\left(P_0P'+P'^2\right)$$

(Neglecting third and higher order fluctuating terms)

$$P^{'}\frac{dP}{dt}=\frac{C}{(K^2+N_0{}^2)}[N_0{}^2P_0P^{'}+N^{'2}P_0P^{'}+2N_0P_0N^{'}P^{'}+N_0{}^2P^{'2}]-\frac{2CN_0{}^3P_0N^{'}P^{'}}{(K^2+N_0{}^2)^2}-D(P_0P^{'}+P^{'2})$$

Taking average on both sides and using $<P^{'}(r)>=0$, $<N^{'}(r)>=0$, we get,

$$<P^{'}\frac{dP}{dt}>=\frac{CN_0{}^2<P'^2>}{(K^2+N_0{}^2)}+\frac{2CK^2N_0P_0<N'P'>}{(K^2+N_0{}^2)^2}-D<P'^2>$$
$$\Rightarrow\frac{1}{2}\frac{d<P'^2>}{dt}=\frac{CN_0{}^2<P'^2>}{(K^2+N_0{}^2)}+\frac{2CK^2N_0P_0<N'P'>}{(K^2+N_0{}^2)^2}-D<P'^2>\tag{7}$$

Similarly, multiplying 2 with N' using 3 and 4 and then after taking average, applying properties of Reynolds average to exchange the derivative and average for $<N'^2>$, (that is, for any real variable u, $<\frac{du}{dt}>=\frac{d<u>}{dt}$), and using $<P^{'}(r)>=0$, $<N^{'}(r)>=0$, we get,

$$\frac{1}{2}\frac{d<N'^2>}{dt}=-\frac{CN_0{}^2<N'P'>}{(K^2+N_0{}^2)}-\frac{2CK^2N_0P_0<N'^2>}{(K^2+N_0{}^2)^2}+D<N'P'>\tag{8}$$

Now we have to find out $\frac{d<N'P'>}{dt}$.

$$
\begin{aligned}
&\frac{d(N'P')}{dt} = N^{'}\frac{dP'}{dt} + P^{'}\frac{dN'}{dt} \\
&\Rightarrow \frac{d(N'P')}{dt} = N^{'}\frac{d(P-P_0)}{dt} + P^{'}\frac{d(N-N_0)}{dt} \\
&\Rightarrow \frac{d(N'P')}{dt} = N^{'}(\frac{dP}{dt} - \frac{dP_0}{dt}) + P^{'}(\frac{dN}{dt} - \frac{dN_0}{dt}) \\
&\Rightarrow < \frac{d(N'P')}{dt} > = < N^{'}(\frac{dP}{dt} - \frac{dP_0}{dt}) + P^{'}(\frac{dN}{dt} - \frac{dN_0}{dt}) > \\
&\Rightarrow < \frac{d(N'P')}{dt} > = < N^{'}\frac{dP}{dt} > + < P^{'}\frac{dN}{dt} > - < N^{'}\frac{dP_0}{dt} > - < P^{'}\frac{dN_0}{dt} > \\
&\Rightarrow \frac{d<N'P'>}{dt} = < N^{'}\frac{dP}{dt} > + < P^{'}\frac{dN}{dt} > \\
&\left(\text{since } < N^{'}\frac{dP_0}{dt} > = 0, < P^{'}\frac{dN_0}{dt} > = 0\right) \\
&\left(\text{Multiplying (1) with } N^{'} \text{ and using (3) and (4) and neglecting third and higher order fluctuating terms, we obtain,}\right) \\
&N^{'}\frac{dP}{dt} = \frac{C}{(K^2+N_0{}^2)}\left(N_0{}^2P_0N^{'} + 2N_0P_0N^{'2} + N_0{}^2N^{'}P^{'}\right) - \frac{2CN_0{}^3P_0N^{'2}}{(K^2+N_0{}^2)^2} - D(P_0N^{'} + N^{'}P^{'})
\end{aligned}
$$

After taking average on both sides of above equation and using $< P^{'}(r) >=0$, $< N^{'}(r) >=0$, we obtain,

$$
< N^{'}\frac{dP}{dt} > = \frac{CN_0{}^2 < N'P' >}{(K^2+N_0{}^2)} + \frac{2CK^2N_0P_0 < N'^2 >}{(K^2+N_0{}^2)^2} - D < N'P' > \tag{9}
$$

Similarly, multiplying (2) with P', using (3) and (4), neglecting third and higher order fluctuating terms and then taking average and using $< P^{'}(r) >=0$, $< N^{'}(r) >=0$, we get,

$$
< P'\frac{dN}{dt} > = -\frac{CN_0{}^2 < P'^2 >}{(K^2+N_0{}^2)} - \frac{2CK^2N_0P_0 < N'P' >}{(K^2+N_0{}^2)^2} + D < P'^2 > \tag{10}
$$

Adding 9 and 10, we get,

$$
\frac{d < N'P' >}{dt} = \frac{CN_0{}^2(< N'P' > - < P'^2 >)}{(K^2+N_0{}^2)} + \frac{2CK^2N_0P_0(< N'^2 > - < N'P' >)}{(K^2+N_0{}^2)^2} + D(< P'^2 > - < N'P' >) \tag{11}
$$

Equations (5), (6) represent time variation of mean terms P_0, N_0, equations (7), (8) represent time variation of variance terms $< P'^2 >, < N'^2 >$ and lastly equation (11) represents time variation of covariance term $< N'P' >$ respectively. Now adding equations (5), (6), we obtain,

$$
\begin{aligned}
&\frac{dP_0}{dt} + \frac{dN_0}{dt} = 0 \Rightarrow P_0 + N_0 = \text{Constant} \\
&\Rightarrow < P + N > = \text{Constant} \Rightarrow \text{Constant} = A \Rightarrow P_0 + N_0 = A
\end{aligned}
$$

Now adding equations (7), (8) and (11), we obtain,

$$
\begin{aligned}
&\frac{1}{2}\frac{d < P'^2 >}{dt} + \frac{1}{2}\frac{d < N'^2 >}{dt} + \frac{d < N'P' >}{dt} = 0 \\
&\Rightarrow < P'^2 > + < N'^2 > + 2 < N'P' > = \text{Constant} = B(say)
\end{aligned}
$$

2 Dimensionless System

we will use the following scaled variables and dimensionless parameters to form a dimensionless system.
Let, $p_0 = \frac{P_0}{A}, n_0 = \frac{N_0}{A}, x = \frac{<P'^2>}{A^2}, y = \frac{<N'^2>}{A^2}, z = \frac{<N'P'>}{A^2}, \kappa = \frac{K}{A}, \varepsilon = \frac{D}{C}, \tau = C\,t$. Therefore, from equation (**??**), we obtain, $p_0 + n_0 = 1$. Similarly, from equation (**??**), we obtain,

$$
x + y + 2z = \beta, \quad \text{where } \beta = \frac{B}{A^2}.
$$

Now substituting the dimensionless parameters and scaled variables into equations (5), (6), (7), (8) and (11) and putting $n_0 = 1 - p_0, z = \frac{(\beta - x - y)}{2}$, we obtain the following dimensionless system of three variables p_0, x, y,

$$\frac{dp_0}{d\tau} = \frac{(1-p_0)^2 p_0}{(k^2+(1-p_0)^2)} + \frac{k^2(1-p_0)(\beta - x - y)}{(k^2+(1-p_0)^2)^2} + \frac{k^2 p_0(k^2 - 3(1-p_0)^2)y}{(k^2+(1-p_0)^2)^3} - \varepsilon p_0,$$
$$\frac{dx}{d\tau} = \frac{2(1-p_0)^2 x}{(k^2+(1-p_0)^2)} + \frac{2k^2(1-p_0)p_0(\beta - x - y)}{(k^2+(1-p_0)^2)^2} - 2\varepsilon x,$$
$$\frac{dy}{d\tau} = -\frac{(1-p_0)^2(\beta - x - y)}{(k^2+(1-p_0)^2)} - \frac{4k^2(1-p_0)p_0 y}{(k^2+(1-p_0)^2)^2} + \varepsilon(\beta - x - y),$$

3 Supplementary Figures

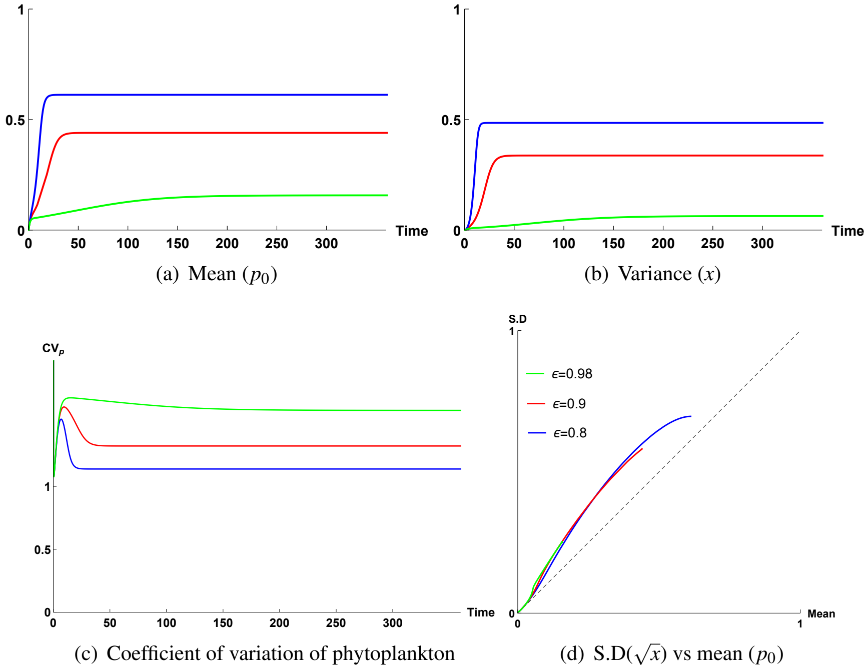

Figure S1. *Time series graph of (a) mean (p_0), (b) variance (x), (c) coefficient of variation of phytoplankton (CV_P), and (d) corresponding parametric plot of coefficient of variation of phytoplankton (CV_P) of the closure model, when ε varies for a fixed β, where $\varepsilon \in (0.35, 1)$, considering total biomass is high, that is, $A = 2\ \mu gNl^{-1}$. The constant parameter values for this simulation are $\kappa = 0.5$ ($K = 1\ \mu gNl^{-1}$, A=2 μgNl^{-1}) and $\beta = 1.2$ for $\varepsilon = 0.8, 0.9, 0.98$ respectively.*

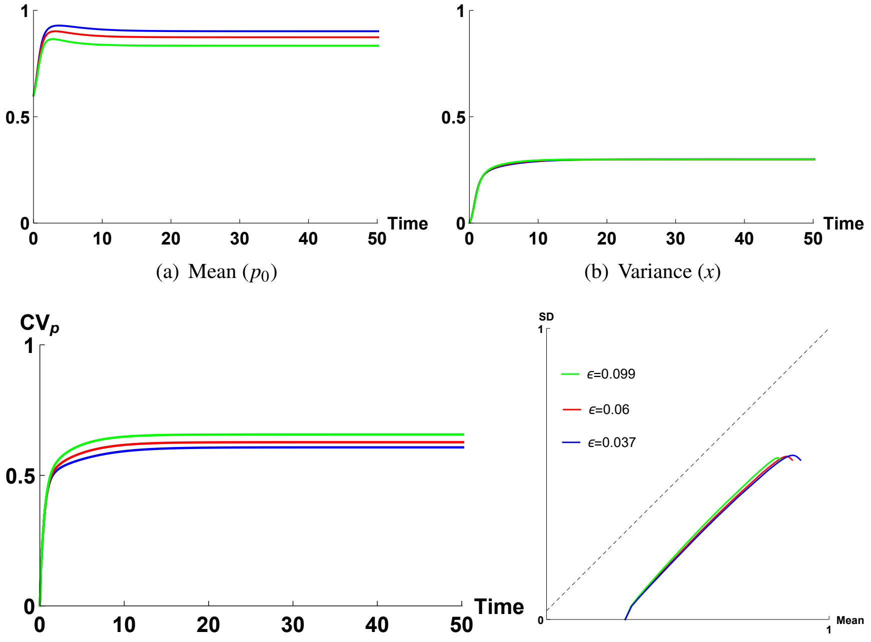

(a) Mean (p_0) (b) Variance (x)

(c) Coefficient of variation (d) S.D (x) vs mean (p_0)

Figure S2. *Time series graph of (a) mean (p_0), (b) variance (x), (c) coefficient of variation of phytoplankton (CV_P), and (d) corresponding parametric plot of coefficient of variation of phytoplankton (CV_P) of the closure model, when ε varies for a fixed β, where $\varepsilon \in (0.035, 0.1)$, considering total biomass is high, that is, $A = 2\ \mu gNl^{-1}$. The constant parameter values for this simulation are $\kappa = 0.5$ ($K = 1\ \mu gNl^{-1}$, $A = 2\ \mu gNl^{-1}$) and $\beta = 0.3$ for $\varepsilon = 0.037, 0.06, 0.099$ respectively.*

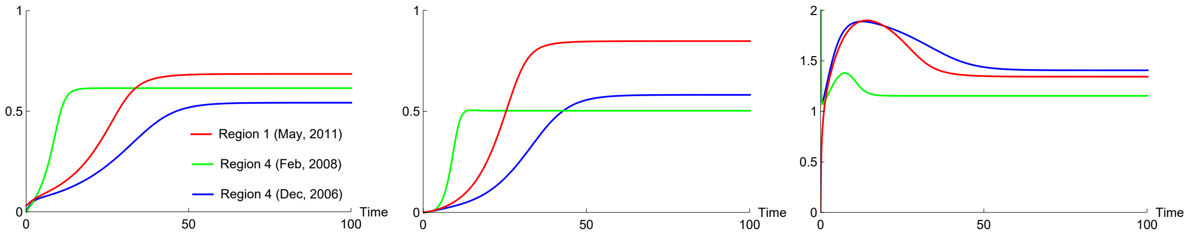

(a) Mean (p_0) (b) Variance (x) (c) Coefficient of variation

Figure S3. *Time series graph of (a) mean (p_0), (b) variance (x), (c) coefficient of variation of phytoplankton (CV_P) of the closure model for Region 4, Region 1 in Dec 2006, Feb 2008, May 2011 respectively. These figures represent how seasonal and regional variations affect CV_P at the depth of 50 meters of Region 4 (inside Tokyo Bay) and at the depth of 200 meters of Region 1, where $\varepsilon \in (0.35, 1)$, considering total biomass of all regions is high, $A = 2\mu gNl^{-1}$. The constant parameter values for this simulation are $\kappa = 0.3$ ($K = 0.6\mu gNl^{-1}$, $A = 2\ \mu gNl^{-1}$) for Region 1 and $\kappa = 0.4$ ($K = 0.8\ \mu gNl^{-1}$, A=2 μgNl^{-1}) for Region 4 and $\beta = 1.6, 1.6, 0.9$ for $\varepsilon = 0.93, 0.95, 0.75$ in May, Dec and Feb at Region 1, Region 4 respectively.*

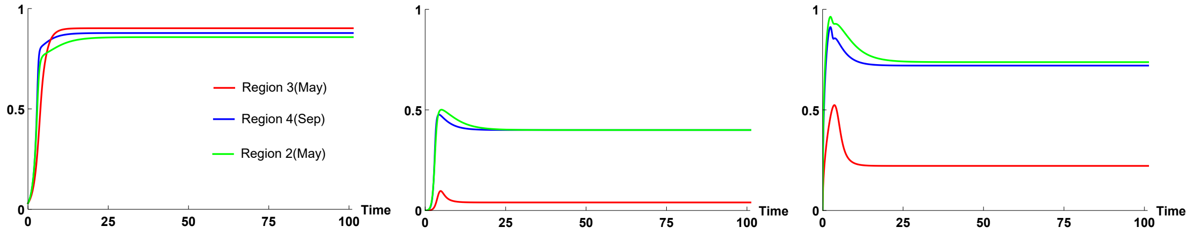

(a) Mean (p_0) (b) Variance (x) (c) Coefficient of variation

Figure S4. *Time series graph of (a) mean (p_0), (b) variance (x), (c) coefficient of variation of phytoplankton (CV_P) of the closure model for Region 4 in Sep 2007 and Region 2, Region 3 in May 2011 respectively. These figures represent how seasonal and regional variations affect CV_P at the depth of 10 meters of Region 3 (mouth of Arakawa river) and at the depth of 50 meters of Region 2, Region 4 (inside Tokyo Bay, different locations) where range of $\varepsilon \in (0.035, 0.1)$, considering total biomass of all regions is high, $A = 2\mu gNl^{-1}$. The constant parameter values for this simulation are $\kappa = 0.5$ ($K = 1\ \mu gNl^{-1}$, $A = 2\ \mu gNl^{-1}$) for Region 2 and Region 3 and $\kappa = 0.4$ ($K = 0.8\ \mu gNl^{-1}$, $A = 2\mu gNl^{-1}$) for Region 4 and $\beta = 0.4, 0.04, 0.4$ for $\varepsilon = 0.075, 0.037, 0.085$ in May 2011, Sep 2007 at Region 2, Region 3 and Region 4 respectively.*

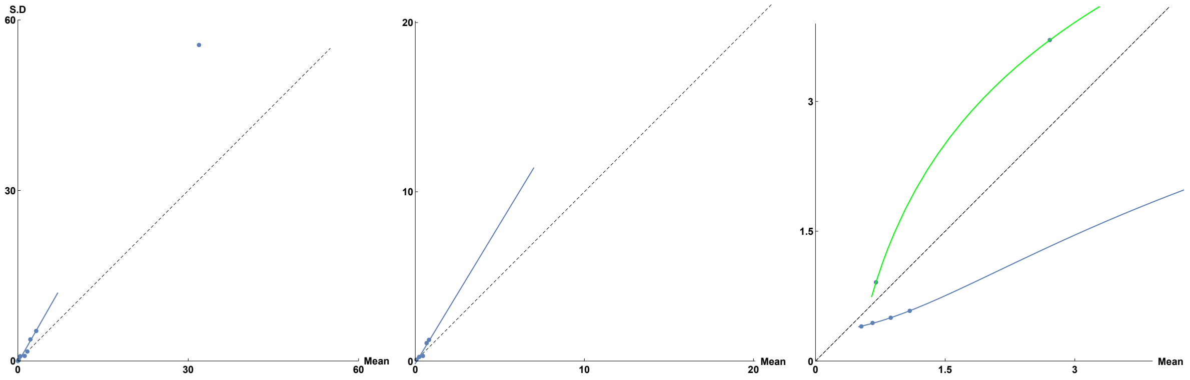

(a) S.D (x) vs Mean (p_0) (b) S.D (x) vs Mean (p_0) (c) S.D (x) vs Mean (p_0)

Figure S5. *Plot of standard deviation versus mean of phytoplankton in (a) spring 1997, (b) summer 1998, in sub-Antarctic of the straits of Magellan ($53^o S$), (c) in four different sampling stations of Funka Bay of Japan between Dec, 1995 and March, 1997 (plotted data is collected from Table**??**).*
